# Immune activation state modulates infant engram expression across development

**DOI:** 10.1126/sciadv.adg9921

**Published:** 2023-11-08

**Authors:** Sarah D. Power, Erika Stewart, Louisa G. Zielke, Eric P. Byrne, Aaron Douglas, Clara Ortega-de San Luis, Lydia Lynch, Tomás J. Ryan

**Affiliations:** ^1^ School of Biochemistry and Immunology, Trinity College Dublin, Dublin, Ireland.; ^2^ Trinity College Institute for Neuroscience, Trinity College Dublin, Dublin, Ireland.; ^3^ Center for Lifespan Psychology, Max Planck Institute for Human Development, Berlin, Germany.; ^4^ Faculty of Psychology and Neuroscience, Maastricht University, Maastricht, Netherlands.; ^5^ Florey Institute of Neuroscience and Mental Health, Melbourne Brain Centre, University of Melbourne, Melbourne, VIC, Australia.; ^6^ Child & Brain Development Program, Canadian Institute for Advanced Research (CIFAR), Toronto, ON, Canada.; ^7^ Brigham and Women’s Hospital, Harvard Medical School, Boston, MA, USA.

## Abstract

Infantile amnesia is possibly the most ubiquitous form of memory loss in mammals. We investigated how memories are stored in the brain throughout development by integrating engram labeling technology with mouse models of infantile amnesia. Here, we found a phenomenon in which male offspring in maternal immune activation models of autism spectrum disorder do not experience infantile amnesia. Maternal immune activation altered engram ensemble size and dendritic spine plasticity. We rescued the same apparently forgotten infantile memories in neurotypical mice by optogenetically reactivating dentate gyrus engram cells labeled during complex experiences in infancy. Furthermore, we permanently reinstated lost infantile memories by artificially updating the memory engram, demonstrating that infantile amnesia is a reversible process. Our findings suggest not only that infantile amnesia is due to a reversible retrieval deficit in engram expression but also that immune activation during development modulates innate, and reversible, forgetting switches that determine whether infantile amnesia will occur.

## Teaser

Infantile amnesia suppresses the expression of memory engram ensembles and is modulated by maternal immune activation experience.

## INTRODUCTION

Infantile amnesia is the rapid forgetting of memories formed during early development and is a largely neglected form of memory loss that seemingly affects the entirety of the human population (*1*, *2*). Not uniquely a human phenomenon, this form of amnesia has been documented in rodents, which shows the forgetting of contextual and fear memories formed during the infant period (*3*–*6*). Little is known about the basic neurobiology of infantile amnesia or its effect on the engram cell ensembles that encode specific memories. Because of the integration of activity-dependent ensemble labeling with optogenetics, it is now possible to investigate whether a memory engram is still present and/or functional in the brain even in the case of amnesia (*7*). Using this methodology, it has been shown that memory recall can be induced after amnesia by optogenetic activation of engram cells in the hippocampus and other brain regions, demonstrating that these memories are not only still present in the brain but also are recoverable (*8*–*11*). This framework provides an opportunity to investigate how development affects the storage and retrieval of early childhood memories. Environmental conditions strongly influence both learning and forgetting rates, but less is known about how forgetting occurs under altered developmental trajectories (*12*). During embryonic and postnatal development, there are periods in which the developing brain has heightened sensitivity to environmental influences (*6*, *13*–*18*). Infantile amnesia has been shown to be preventable through postnatal pharmacological interventions using γ-aminobutyric acid agonists (*19*–*21*) and corticosteroids (*6*) or ectopic administration of neurotrophins (*22*), but the relevance of these plasticity switches in connecting environmental experiences with memory function across natural development is unknown. Events during embryonic development such as the activation of the immune system, and subsequent cytokine release, are known to induce altered developmental trajectories associated with autism spectrum disorder (ASD) and schizophrenia, but the impact on engram function has not been explored (*13*, *23*–*26*). Here, we sought to identify naturally occurring variation in infantile amnesia caused by the developmental experience of the animal, and then investigate the effects on engram cell function.

## RESULTS

### Maternal immune activation during the embryonic period alleviates infantile amnesia in male offspring

**Fig. 1.**
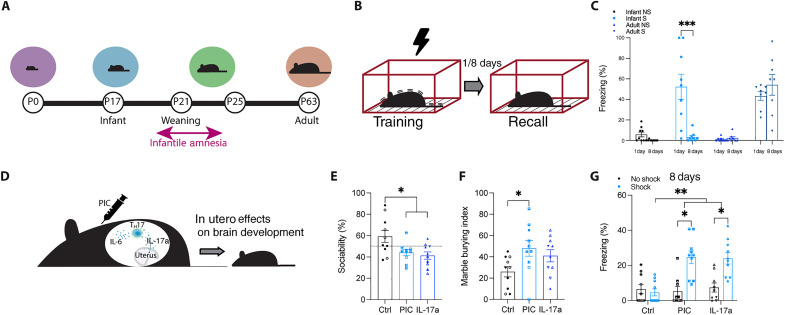
MIA male offspring do not demonstrate infantile amnesia in a contextual fear paradigm. (**A**) Developmental trajectory of infant mice. (**B**) Behavioral schedule. The black lightning symbol represents foot shocks. Mice are trained using contextual fear conditioning (CFC) in context A and tested for recall 1 or 8 days later. S, shock; NS, no shock. (**C**) Memory recall in context A. Adult (P63) C57BL/6 J male mice (*n* = 8) froze significantly more in context A than no shock controls (*n* = 8) both 1 and 8 days after training. Infant (P17) C57BL/6 J male mice (*n* = 9) froze significantly more than no shock controls during recall 1 day after training. No significant difference in freezing between groups (*n* = 9) when tested for recall 8 days after training. (**D**) Representative diagram of maternal immune activation (MIA) in pregnant dams. The syringe indicates poly(I:C) (PIC) injection at E12.5. (**E**) Social preference index (% time spent exploring social stimulus out of total object investigation time) of MIA adult C57BL/6 J male offspring (*n* = 10). (**F**) Marble-burying behavior of MIA adult C57BL/6 J male offspring (*n* = 10). The marble burying index is plotted on the *y* axis. (**G**) Memory recall in male C57BL/6 J MIA offspring in context A after CFC at P17 (*n* = 10). *P < 0.05, **P < 0.01, and ***P < 0.001 were calculated by (C) two-way analysis of variance (ANOVA) or (E to G) nested ANOVA with Bonferroni post hoc tests. Data are presented as means ± SEM. IL-6, interleukin-6; IL-17a, interleukin-17a; T_H_17, T helper 17.

Using a contextual fear conditioning (CFC) paradigm (Fig. 1, A and B), we trained and tested infant (P17) (*27*–*30*) and adult (P63) mice for fear memory recall 1 or 8 days after training (Fig. 1C). At both time points, the experimental adult shock group showed significantly more freezing than the no shock control group (Fig. 1C). Infant mice tested for memory recall 1 day following training showed significantly higher levels of freezing compared to the control group (Fig. 1C), while the group tested 8 days after training showed robust infantile amnesia, with similar levels of freezing to the no shock controls (Fig. 1C). Consistent with the literature, infant mice demonstrate forgetting as early as 1 week after training, while adult mice show continuous memory retention (*4*, *31*).

We tested whether altering external environmental influences, such as environmental enrichment during infancy and the time of weaning from maternal care, affected the occurrence of infantile amnesia (fig. S1). We next looked at the effect of altered developmental trajectories resulting from embryonic challenges. During pregnancy, exposure to pathogens or inflammation during critical periods of brain development [maternal immune activation (MIA)] has been shown to alter postnatal brain growth and cognitive development through the release of pro-inflammatory cytokines such as the interleukin-6 (IL-6) and IL-17 families (*32*). Delivery of the viral-mimetic compound, polynosinic:polycytidylic acid [poly(I:C)], at E12.5 is a well-established method for the experimental induction of MIA in rodents (*33*–*35*). More recently, the maternally derived cytokine, IL-17a, has been shown to mediate the effect of poly(I:C) effect on MIA and is sufficient to induce an altered brain state in offspring (*13*, *17*, *23*, *24*, *36*). Here, we used the administration of either poly(I:C), or recombinant IL-17a, as two distinct methods to stimulate MIA in pregnant dams at E12.5 in C57BL/6 J mice (Fig. 1D). To first validate our method, we measured changes in IL-17a blood serum after injection of poly(I:C) or phosphate-buffered saline (PBS) in female mice (fig. S2A). Injection of poly(I:C) resulted in an increase in IL-17a in the blood serum compared to PBS controls. In line with published studies, male, but not female, offspring from pregnant dams treated with poly(I:C) or IL-17a demonstrated repetitive behavior and deficits in social behavior, behavioral phenotypes indicative of ASD (Fig. 1, E and F, and fig. S2, B to G) (*13*, *23*, *24*). To test whether MIA causes any effect on memory retention, we next trained infant offspring from MIA dams using CFC at P17 (Fig. 1G and fig. S2, H to J). When tested 1 day after training, all cohorts showed similar levels of freezing, irrespective of sex (fig. S2, H and I). However, male, but not female, offspring from pregnant dams treated with poly(I:C) or IL-17a showed retention of infant memory, like that of adult mice, when tested 8 days after training (Fig. 1G and fig. S2J). This effect was also true when MIA male offspring were tested for recall of a CFC memory 15 days after training (fig. S2K). Regardless of whether male MIA offspring were tested for recall 8 or 15 days after training, the fear memory was retained. Furthermore, the effect was sex-specific (interaction, *P* = 0.0119) (fig. S2L). This represents an unknown phenomenon in which male, but not female, offspring from poly(I:C)-injected dams do not demonstrate infantile amnesia for contextual fear memory. This phenomenon was also witnessed in offspring from dams injected with IL-17a, indicating the mediating role of IL-17a signaling.

### MIA male offspring show an increase in engram labeling and spine density in the DG

**Fig. 2.**
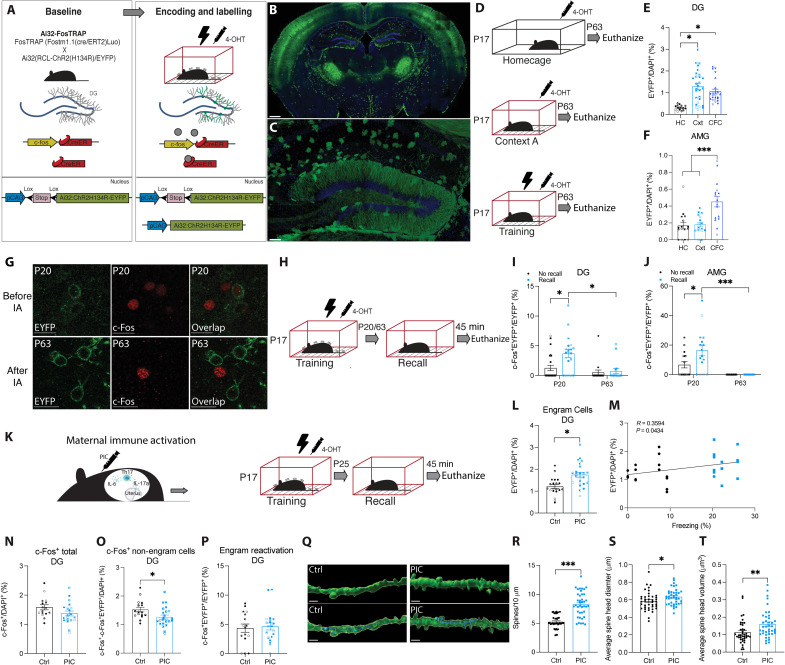
Natural reactivation of engram cells for an infant-encoded memory. (**A**) Engram labeling method in Ai32-FosTRAP mice. (**B**) Whole-brain engram labeling of an infant engram. Scale bar, 400 μm. (**C**) Dentate gyrus (DG) engram cells (ChR2-EYFP^+^ green) in Ai32-FosTRAP mice. Scale bar, 100 μm. (**D**) Behavioral schedule for engram labeling of the home cage (HC), a context (Cxt), or contextual fear memory (CFC) in P17 Ai32-FosTRAP mice. The black lightning symbol represents foot shocks. The syringe symbol represents 4-hydroxytamoxifen (4-OHT) injection 2 hours after training. (**E** and **F**) ChR2-EYFP^+^ cell counts in the DG (*N* = 4 to 6, *n* = 4) and amygdala (AMG) (*N* = 4, *n* = 4). (**G**) Example images of DG ChR2-EYFP^+^ (green) and c-Fos^+^ (red) cell counts after recall at P20 and P63. IA, infantile amnesia. Scale bars, 20 μm. (**H** and **K**) Behavioral schedule. (**I** and **J**) Engram reactivation (c-Fos^+^ChR2-EYFP^+^/ChR2-EYFP^+^) in the DG (*N* = 4 to 6, *n* = 4) and AMG (*N* = 4, *n* = 4) after recall or no recall (home cage) at P20 and P63. (**L**) ChR2-EYFP^+^ cell counts in the DG in MIA male offspring (*N* = 5 to 6, *n* = 4). (**M**) Scatter plot of the relationship between the number of EYFP^+^/DAPI (4′,6-diamidino-2-phenylindole) cells and freezing behavior (%) (*N* = 8, *n* = 4). (**N**) c-Fos^+^ cell counts in the DG (*N* = 4 to 5, *n* = 4). (**O**) c-Fos^+^ cell counts in non-engram cells the DG (*N* = 10, *n* = 4). (**P**) c-Fos^+^ChR2-EYFP^+^/ChR2-EYFP^+^ cell counts in the DG (*N* = 4 to 5, *n* = 4). (**Q**) Engram cell dendritic spines in the DG of MIA male offspring after recall at P25 (*N* = 4, *n* = 10). Scale bars, 40 μm. (**R**) Spine density per 10 μm. (**S**) Average dendritic spine head diameter (in micrometers). (**T**) Average dendritic spine head volume (in square micrometers). *P < 0.05, **P < 0.01, and ***P < 0.001 calculated by (E, L, and N to P) nested Student’s *t* test, (R to T) Student’s *t* test, (M) Pearson’s correlation, or (F, I, and J) nested ANOVA with Bonferroni post hoc tests. Data are presented as ±SEM.

We characterized and used a Cre-based engram labeling strategy for whole-brain tagging of engrams cells in infant mice by crossing a *Fos*^CreER^ (FosTRAP) transgenic line that expresses iCre from an inducible c*-fos* promoter with an Ai32 transgenic mice express channelrhodopsin-2/enhanced yellow fluorescent protein (ChR2-EYFP) (Fig. 2, A to C) (*37*, *38*). Intraperitoneal (IP) injection of the tamoxifen metabolite, 4-hydroxytamoxifen (4-OHT), allows iCre to recombine the ChR2-EYFP transgene (*39*, *40*). Using this engram labeling method, ChR2-EYFP expression can be detected in engram cells 3 days after 4-OHT injection. First, to ensure that infant mice still show memory retention at P20, we trained infant mice using CFC at P17 and tested them for memory recall 3 days later (P20) (fig. S3A). Infant mice trained at P17 show similar memory retention for a fear memory when tested 1 or 3 days after training (fig. S3, B and C). We evaluated the efficiency of the Ai32-FosTRAP engram labeling system in P17 (infant) and P42 mice by quantifying the number of ChR2-EYFP^+^ cells in brain regions associated with context and fear memory, including the dentate gyrus (DG), amygdala (AMG), retrosplenial cortex (RSC), and the periaqueductal gray (PAG) at P63 for a home cage, contextual, or contextual fear memory (Fig. 2, D to F, and fig. S4). Exposure to context A resulted in an activity-dependent increase in engram labeling (EYFP^+^ cells) in the DG at both P17 (Fig. 2E) and P42 (fig. S4B). Similarly, there was an activity-dependent increase in EYFP^+^ labeling in the AMG after CFC at both P17 (Fig. 2F) and P42 (fig. S4C). These results demonstrate that the Ai32-FosTRAP line is an efficient engram labeling system for activity-dependent engram labeling in both infant and adult mice.

We next investigated whether engram cells formed at the time of infant memory encoding are reactivated by exposure to training cues (Fig. 2H). Cellular activation of engram cells was examined by quantifying the number of EYFP^+^ c-Fos^+^ cells before (P20) and after (P63) the infantile amnesia period across multiple brain regions (Fig. 2G and figs. S5 and S6). Natural recall cues result in above chance EYFP^+^ and c-Fos^+^ overlap in the DG and AMG at P20 (Fig. 2, I and J, and fig. S5, D and G), an effect that was not seen at P63 (Fig. 2, I and J, and fig. S6, D and G). We next compared engram reactivation after natural recall for contextual fear memory in male offspring from MIA dams (Fig. 2K). Male, but not female, offspring from MIA dams have an increased number of EYFP^+^ engram cells in the DG compared to controls (Fig. 2L and fig. S7, A and B), indicating an altered engram ensemble also reported for a genetic model of ASD (*41*). Furthermore, there was a positive correlation between freezing behavior (%) and the total number of engram cells (EYFP^+^) in the DG of male MIA offspring [*r*(30) = 0.36, *P* = 0.043; Fig. 2M]. A larger engram size was related to an increase in freezing behavior during recall of contextual fear memory in P25 male MIA offspring. Although no difference was seen between groups in the total level of cell activation (c-Fos^+^) (Fig. 2N), MIA male offspring demonstrated a decrease specifically in non-engram cell activation in the DG during recall (Fig. 2O). No difference was seen in the relative level of engram reactivation between groups as both demonstrated an above chance level of EYFP^+^ and c-Fos^+^ overlap in the DG (Fig. 2P and fig. S7C). While prior research has examined alterations in dendritic spines within the cortex of infant MIA offspring (*42*), we sought to investigate the specific modifications in dendritic spines related to engram cells within the DG of MIA male offspring after recall (Fig. 2Q). Dendritic spine analysis indicated that engram cells in MIA offspring had significantly increased dendritic spine density (Fig. 2R) as well as larger spine head diameter and volume relative to control animals at P25 (Fig. 2, S and T). Thus, a combination of larger engram size, lower non-engram cell activation, and increased dendritic plasticity may contribute to enhanced functional engram expression of infant engrams in MIA offspring.

### Optogenetic stimulation of an infant engram allows for acute and permanent reinstatement of the forgotten memory

**Fig. 3.**
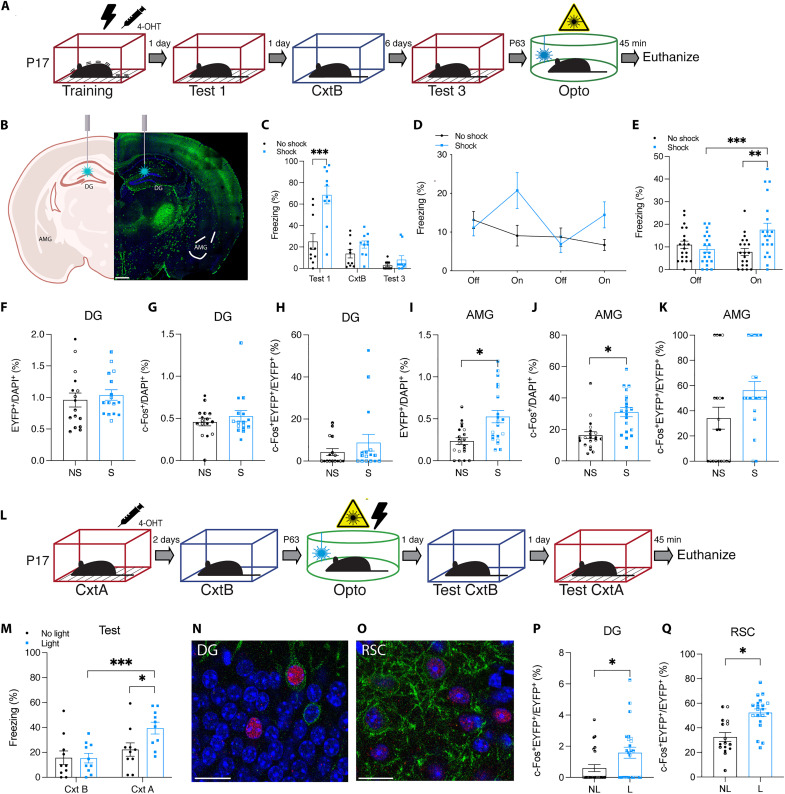
Artificial updating of infant-encoded engram cells permanently reinstates a “lost” infantile memory. (**A**) Behavioral schedule for optogenetic reactivation of infant DG engram cells in adulthood. (**B**) Representative image of optogenetic stimulation of DG engram cells in Ai32-FosTRAP–labeled mice. Scale bar, 400 μm. (**C**) Freezing levels of infant mice (*n* = 10) during natural memory recall. (**D**) Memory recall in context C (engram reactivation) with light-off and light-on epochs. (**E**) Freezing for the two light-off and light-on epochs averaged. (**F** and **I**) ChR2-EYFP^+^ cell counts in the DG (*N* = 4, *n* = 4) and AMG (*N* = 5, *n* = 4) after optogenetic stimulation at P63. (**G** and **J**) c-Fos^+^ cell counts in the DG and AMG. (**H** and **K**) c-Fos^+^ChR2-EYFP^+^/ChR2-EYFP^+^ cell counts in the DG and AMG. (**L**) Behavioral schedule for artificial updating of an infant engram. (**M**) Freezing levels during recall test in context B and context A (*n* = 10). The experimental light group froze significantly more in context A. (**N** and **O**) Histological representation of engram reactivation in the (N) DG and (O) RSC during recall in context A. DAPI^+^ (blue), ChR2-EYFP^+^ (green), c-Fos^+^ (red). Scale bars, 20 μm. (**P** and **Q**) DG (*N* = 6, *n* = 4) and RSC (*N* = 4 to 5, *n* = 4) c-Fos^+^ChR2-EYFP^+^/ChR2-EYFP^+^ cell counts after recall in context A. NL, no light; L, light. *P < 0.05, **P < 0.01, and ***P < 0.001 calculated by (F to K, P, and Q) nested Student’s *t* test or (C, E, and M) two-way ANOVA with Bonferroni post hoc tests. Data are presented as ±SEM.

To investigate the functionality of memories after naturally occurring infantile amnesia, we tested the behavioral effect of optogenetically stimulating infant-labeled DG engram cells in adult mice (Fig. 3A). Engram cells for context A were labeled in Ai32-FosTRAP mice at P17 using CFC (Fig. 3, A to C). Although both cohorts were placed in context A, only the experimental group received foot shocks (S). At P63, both cohorts were placed in context C for a 12-min test session where they received four 3-min epochs of blue light on or off. During the light-off epochs, both groups showed background levels of freezing (Fig. 3D). The experimental group showed significantly higher levels of freezing during the light-on epochs (Fig. 3, D and E). Despite the same number of EYFP^+^ cells being labeled in the DG (Fig. 3F), this light-induced freezing was not observed for animals that underwent the same behavioral paradigm but did not receive any footshocks (NS) at P17 (Fig. 3, D and E). Meanwhile, the stimulation of a neutral memory, labeled in infancy, does not result in freezing behavior (fig. S8, A to C). We extended this paradigm by optogenetically stimulating infant-labeled CA1 engram cells in adult mice (fig. S8D) (*8*, *43*, *44*). A previous study demonstrated that the application of the standard 20-Hz protocol for activating CA1 engram cells did not effectively induce memory recall (*43*). However, implementing a 4-Hz protocol to stimulate CA1 engram cells elicited comparable improvements in recovering memory deficits from anisomycin-induced amnesia (*8*). Optogenetic stimulation at 4 Hz (fig. S8, H and I), but not 20 Hz (fig. S8F), of infant-labeled engram cells in the CA1 of adult mice resulted in light-induced freezing. On the basis of these data, “lost” infantile memories can be acutely recalled by optogenetic stimulation in either the DG or CA1.

Although optogenetic stimulation of a forgotten infant memory in adulthood resulted in a specific behavioral response, it is unclear how the information survives. We investigated the connectivity between engram cells in downstream regions, after infantile amnesia, by histologically assessing engram cell reactivation following optogenetic stimulation of upstream engram cells (Fig. 3, F to K) (*8*, *45*). The resulting c-Fos^+^ counts were equivalent in the hippocampus after light activation of a CFC (shock, S) or a contextual (no shock, NS) memory (Fig. 3G). In contrast, c-Fos^+^ cell counts were significantly higher in the AMG after light activation of an infant CFC memory (Fig. 3J), demonstrating an increase in both AMG activity and the behavioral expression of freezing. Light activation of DG engram cells resulted in an above-chance c-Fos^+^ and EYFP^+^ overlap in the DG (Fig. 3H and fig. S9B) and crucially in other downstream brain regions including the AMG (Fig. 3K and fig. S9C), RSC (fig. S9, F and G) and PAG (fig. S9, J and K). These results were consistent for light activation of both a CFC (shock, S) and a contextual (no shock, NS) memory. These results demonstrate that the connectivity pattern of the engram survives infantile amnesia and persists into adulthood, and optogenetic stimulation of a lost infantile memory is sufficient to reactivate the functional connectivity patterns. As a positive control, we optogenetically stimulated an engram encoded after the infantile amnesia period (P29) (fig. S10A). By selecting this specific stage of development for labeling, we were able to target a memory engram that was not only formed post-infantile amnesia and therefore retained over a long delay (fig. S3, D and E) but also underwent subsequent developmental processes. Optogenetic stimulation of an engram encoded after (P29) the infantile amnesia period also increased engram reactivation to above chance levels in the DG, AMG, RSC, and PAG (fig. S10, B to Q).

Since optogenetic stimulation results in memory recall under artificial conditions, we sought to permanently reinstate an infant memory by adopting an optogenetic induction procedure to induce plasticity in engram cells (fig. S11, A and B) (*8*, *43*). Infant mice can form purely contextual memories that can be updated with shock information (fig. S11, C and D), provided that updating occurs before the onset of infantile amnesia (fig. S11, E and F). At P63 and in a novel context, we optogenetically stimulated DG engram cells for context A, originally encoded at P17, while simultaneously delivering footshocks to create a false association of an infant context engram with adult shock experience (Fig. 3L). Animals subsequently froze significantly more when exposed to context A even though they were shocked in context C (Fig. 3M). This increased freezing was not due to generalization since the control group did not freeze in context B. There was also an increased level of overlap of c-Fos^+^ and EYFP^+^ cells in the DG (Fig. 3, N and P, and fig. S12D) and RSC (Fig. 3, O and Q) after exposure to context A, demonstrating that permanent engram reinstatement was also reflected at a cellular level (fig. S12, B to O). This histological result demonstrates that engram cells active at the time of encoding were reactivated during adult exposure to context A. Together, these findings show that the plasticity that accompanies the targeted updating of an infant engram restores the natural accessibility of that engram to the appropriate perceptual cues and that context specificity is maintained into adulthood.

### MIA offspring do not show infantile amnesia for spatial or object memories and these memories can be optogenetically activated in control offspring

**Fig. 4.**
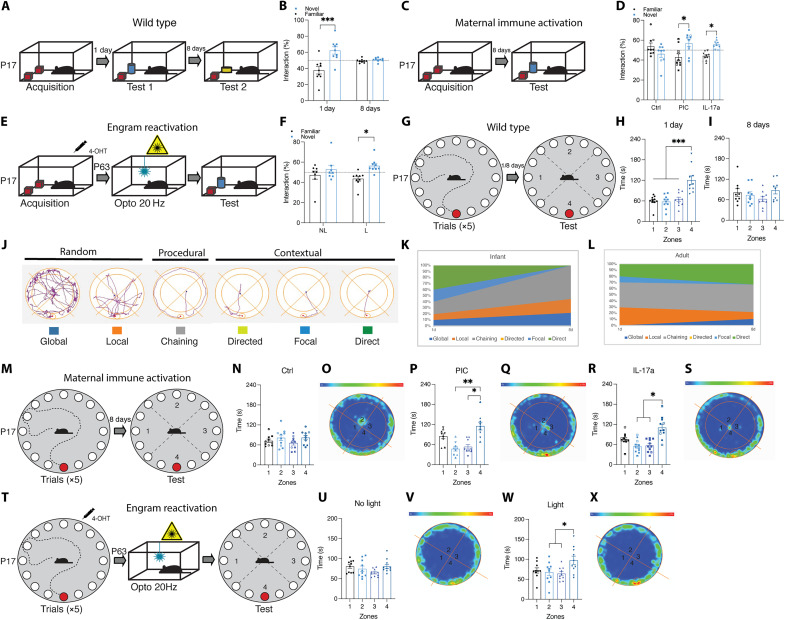
Infantile amnesia for context, object, and maze memory. (**A** and **C**) Behavioral schedule for novel object recognition. (**B**) Interaction index (%) of infant C57BL/6 J mice during novel object recognition test 1 or 8 days after acquisition (*n* = 9). (**D**) Interaction index of MIA male infant offspring (*n* = 9 to 10). (**E**) Behavioral schedule for DG optogenetic reactivation of an infant-encoded engram for an object. (**F**) Interaction index of adult mice after optogenetic stimulation (*n* = 9 to 10). (**G**) Behavioral schedule for the Barnes maze in infant C57BL/6 J mice. (**H** and **I**) Time spent in each zone during the test (H) 1 or (I) 8 days after training. (**J**) Example representations of navigational search strategies. (**K** and **L**) Infant and adult search strategies during testing 1 and 8 days after training. (**M**) Behavioral schedule for the Barnes maze in infant C57BL/6 J male MIA offspring. (**N**, **P**, and **R**) Time spent in each zone by MIA male offspring during test 8 days after training (*n* = 9 to 11). (**O**, **Q**, and **S**) Heatmap analysis of probe test in (O) Ctrl, (Q) PIC, and (S) IL-17a male MIA offspring. (**T**) Behavioral schedule for DG optogenetic reactivation of an infant-encoded engram for the Barnes maze. (**U** and **W**) Time spent in each zone during the test (*n* = 8 to 9). (**V** and **X**) Heatmap analysis of probe test in (V) no light and (X) light groups. *P < 0.05, **P < 0.01, and ***P < 0.001 calculated by (H, I, U, and W) ANOVA, (D, N, P, and R) nested ANOVA, (B) two-way repeated-measures ANOVA, or (F) two-way ANOVA with Bonferroni post hoc tests. Data are presented as ±SEM.

Although fear conditioning is a robust assay, we wanted to further expand our knowledge by testing the relevance of infantile amnesia to other types of memory. To do this, we next looked at this form of forgetting in a novel object recognition behavioral paradigm (Fig. 4A). Although studies have looked at novel object location in infant rats, there has been no clear demonstration of infantile amnesia for novel object recognition in mice at this stage of development (*46*, *47*). Infant mice demonstrated rapid forgetting for novel object recognition tasks, following the developmental trend like that of other forms of infant-encoded memories (Fig. 4B). Both adult and infant cohorts spent more time exploring a novel object relative to a familiar object when tested 1 day after acquisition, demonstrating that a long-term memory was formed (Fig. 4B and fig. S13, A to C). When tested again 8 days later, only the adult cohort spent significantly more time exploring the novel object (Fig. 4B and fig. S13C). We then investigated whether male offspring from MIA dams show infantile amnesia for object memory (Fig. 4C). When tested for object memory retention, male offspring from MIA dams spent significantly more time with the novel object 8 days after acquisition (Fig. 4D and fig. S13E). Like contextual fear memory, male offspring from poly(I:C)- or IL-17a–injected dams do not show infantile amnesia for an object memory.

We then investigated whether it was possible to rescue a lost infant object memory by optogenetic stimulation of infant-labeled engram cells (Fig. 4E and fig. S13F). On the test day, animals that received light stimulation 3 min before the test demonstrated a preference for the novel object (Fig. 4F). While animals that did not receive light stimulation before the test spent the same amount of time exploring both the novel and familiar object. Thus, these object memory engrams survive infantile amnesia, can be retrieved artificially, and their forgetting can be prevented by MIA.

Spatial and object tasks in rodents are useful for the development of parallel, translational object/context and maze learning assays in human behavior experiments (*48*). To expand our analysis of infantile amnesia to an active, maze-based task, we probed memory for a spatial location in infant and adult C57BL/6 J mice using a cued version of the Barnes maze (Fig. 4G and fig. S14G). All cohorts sufficiently learned the escape hole’s location during training over five sessions, evidenced by a gradual decrease in the latency, and distance traveled, to reach the escape hole (fig. S14, A, B, H, and I). Unlike adults, infant mice demonstrated memory retention for the location of the escape hole when tested 1 day, but not 8 days, later (Fig. 4, H and I, and fig. S14, C to F and J to O). To the best of our knowledge, this is the first demonstration of infantile amnesia using the Barnes maze paradigm. We assessed memory content profiling changes in navigational strategies taken by the animal during the task (Fig. 4J). Navigational strategies can be categorized as random, procedural, or contextual (*49*–*51*). Infant mice predominantly used contextual search strategies when tested for spatial memory on the Barnes maze 1 day after training (Fig. 4K). However, during the 8-day probe trial, infant mice show a switch in navigational strategy, moving to procedural and random strategies (Fig. 4K). This adaptation in navigational search strategies reflects the forgetting of the location of the escape platform. In contrast, adult mice show similar search patterns both 1 and 8 days after training (Fig. 4L). Like other forms of memory, we also found that male offspring from MIA models do not show infantile amnesia for a spatial navigation task (Fig. 4, M to S).

By labeling the engram ensembles for the location of the escape platform in the Barnes Maze in infants, we next investigated whether DG optogenetic stimulation of the engram in adulthood results in the location of the escape hole (Fig. 4T). The experimental light group received 3-min light stimulation before being placed on the Barnes maze for a probe test. The experimental light group spent significantly more time in the zone where the escape hole should be when compared to zones 2 and 3 (Fig. 4W). Although there was no significant difference in the total time spent between zone 4 and zone 1, heatmap analysis of the mean time spent in each location showed a concentration around the location where the escape hole previously was located during training (Fig. 4X). No difference was seen in the total time spent between zones for the no-light control group (Fig. 4, U and V). In a separate experiment (fig. S14P), a different cohort of mice was trained where the location of the escape hole during memory encoding differed from experiment Fig. 4T. The location of the escape hole during encoding did not affect the successful recall of the location of the escape hole after light stimulation (fig. S14, Q to T). Optogenetic stimulation of EYFP-expressing neurons in the DG (neurons labeled during infant encoding of the Barnes maze memory) allows for recall of the location of the escape hole in the Barnes maze.

### IL-17a is necessary for the maternal poly(I:C) effect on infantile amnesia in offspring

**Fig. 5.**
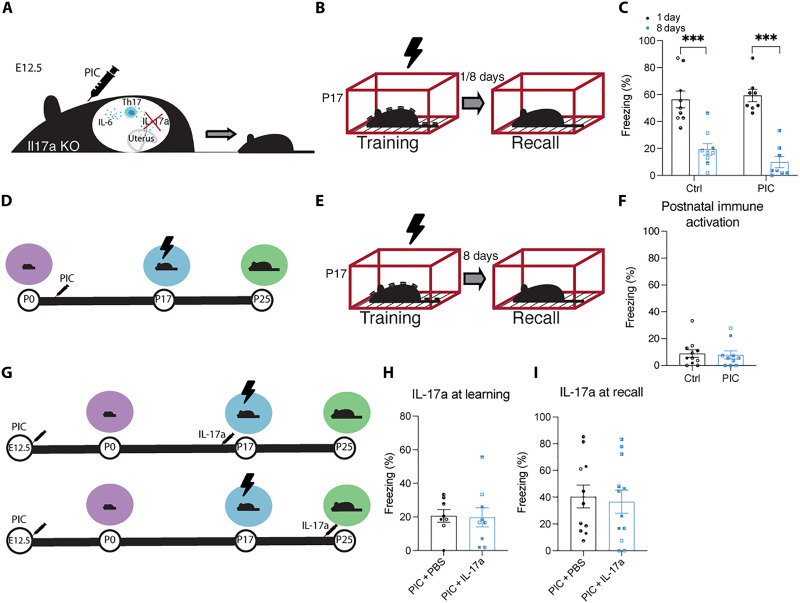
IL-17a is required for MIA effects on infantile amnesia. (**A**) Representative diagram of MIA in *Il17a* KO mice. (**B**) Behavioral schedule for CFC in *Il17a* KO male mice. (**C**) Freezing levels of male *Il17a* KO infant mice during recall 1 or 8 days after training. (**D**) Behavioral schedule for postnatal immune activation in C57BL/6 J infant mice. The syringe symbol represents PIC injection at P3, P7, and P14. (**E**) Behavioral schema for CFC. (**F**) Freezing levels of infant male mice when tested 8 days after training. (**G**) Behavioral schedule for IL-17a injection 3 hours before memory encoding (P17) or recall (P25) in MIA C57BL/6 J offspring. (**H** and **I**) Freezing levels of infant male mice when tested 8 days after training. *P < 0.05, **P < 0.01, and ***P < 0.001 calculated by (F, H, and I) nested Student’s *t* test or (C) nested ANOVA with Bonferroni post hoc tests. Data are presented as ±SEM.

Having established that immune activation and direct administration of the downstream cytokine Il-17a are sufficient to induce altered infantile amnesia, we next sought to test whether Il-17a is necessary for this effect to occur. We injected pregnant *Il17a* knockout (KO) dams with poly(I:C) at E12.5 (Fig. 5A). Adult male *Il17a* KO mice showed normal memory retention for a CFC memory when trained at P63 (fig. S15). This suggests that the *Il17a* genotype does not affect long-term memory retention in adult *Il17a* KO mice. Adult male and female offspring from MIA *Il17a* KO dams did not demonstrate repetitive behavior (fig. S16E) or deficits in social behavior (fig. S16, A to D). Crucially, infant offspring showed normal infantile amnesia for a CFC memory at P25 (Fig. 5, B and C). Preventing maternal IL-17a signaling, using a genetic model, occluded the MIA effect on infantile amnesia in the offspring. Therefore, the behavioral effect seen following MIA is specifically dependent on IL-17a signaling. We investigated whether altering the timing of immune activation, from maternal to postnatal, would have the same effect on memory retention (Fig. 5D) (*52*). Postnatal immune activation in C57BL/6 J male mice did not affect infantile amnesia, indicating that there is a critical window during prenatal development where IL-17a and immune activation can affect a mammal’s propensity for infantile amnesia (Fig. 5, E and F). Induction of an inflammatory response in MIA offspring has been shown to temporarily rescue deficits in sociability in ASD (*23*). Therefore, we investigated whether we could reverse the effect of MIA on infantile amnesia in the offspring through the administration of IL-17a at the time of memory encoding (P17) or memory recall (P25) (Fig. 5G). MIA male offspring were injected with IL-17a 3 hours before CFC (Fig. 5H) or the recall test (Fig. 5I). Regardless of whether injected with IL-17a or PBS, MIA male infant offspring showed normal memory retention for a CFC memory 8 days after training (Fig. 5, H and I), indicating that the effect of MIA on infantile amnesia cannot be reversed through acute IL-17a administration.

## DISCUSSION

We provide an integrative set of analyses that triangulates infantile amnesia, engram expression, and immune activation state in utero. Together, our findings indicate that infantile amnesia, for a wide range of memory types, and engram cells as the substrate of memory can be modulated by the immunological experiences of the subject during embryonic development. The effects of MIA preserve the natural retrievability of infant engrams across postnatal development. Furthermore, we find that MIA directly modulates engram plasticity at a structural level, altering engram ensemble size and engram dendritic spine density. In addition, engram reactivation shows greater potential specificity under MIA as non-engram cell activity is significantly reduced during recall. This, in turn, may be reflective of a reduction in the interference of competing ensembles (*53*, *54*). Furthermore, our data show that many types of infant memories are naturally suppressed during development, but that their engrams persist and can be acutely activated in adulthood by direct optogenetic stimulation (*31*). Last, we show that the downstream cytokine, IL-17a, is both sufficient and necessary for the effect of MIA on infantile amnesia.

These findings demonstrate that optogenetic reactivation of an infant memory is not specific to fear conditioning, as memories for more complex navigational and recognition tasks can also be rescued. Furthermore, artificially updating an engram restored natural access to the target memory at both a behavioral and cellular level, reversing infantile amnesia. The specific connectivity patterns between engram cells, formed during infancy across distributed brain regions, also remain intact into adulthood. This kind of distributed engram connectivity pattern has been shown to survive other kinds of amnesia and may account for specific stored information to be retained in the brain (the engram itself) (*8*, *9*, *45*, *55*–*58*).

On the basis of our analysis and others, infantile amnesia seems to be an innately driven form of natural forgetting and is not modulated by specific environmental experiences during postnatal development. Immune activation in utero results in the development of an altered brain state where the capacity to form engrams is altered, and infantile amnesia does not occur postnatal immune activation does not seem to affect infantile amnesia, making it plausible that an innate predetermined forgetting switch is fixed from birth. However, interactions between MIA and genetic risk factors for ASD might allow for postnatal inflammation to alter forgetting rates (*52*, *59*).

Our findings suggest that the brain state that permits infantile amnesia is absent in immunological models of ASD, and these findings are reminiscent of transgenic fly experiments and retrospective human studies that have provided evidence for improved long-term memory retention in cases of ASD (*60*, *61*). It is conceivable that the MIA offspring’s brain state may mirror that of precocial mammals, who do not seem to show infantile amnesia (*4*, *62*). Infantile amnesia may represent a genetically canalized form of natural forgetting that can be modulated by developmental plasticity during critical periods. Future studies should determine the nature of the switching mechanism that determines when infantile amnesia occurs, its interaction with the immune system, and its reversible effect on engram cell function and engram ensemble expression. In the future, MIA models may offer opportunities for translational studies of memory and forgetting across the life span in rodents and humans (*63*).

## MATERIALS AND METHODS

### Experimental design

The objective of this study was to investigate how memories are stored in the brain throughout development, specifically focusing on infantile amnesia and its modulation by immune activation. Mice were randomly assigned to experimental groups with littermate controls or otherwise relevant controls. During behavioral testing, investigators were blinded to the treatment conditions in the experimental groups. Histological analysis was conducted individually for each mouse using nested analysis, and the same approach was applied to MIA experiments where analysis was performed per litter. Throughout all experiments, data collection and analysis were conducted blindly.

### Subjects

For wild-type experiments, the C57BL/6 J substrain C57BL/6JOlaHsd was used. The Ai32-FosTRAP mice were generated by crossing FosTRAP (*37*, *38*) mice with Ai32(RCL-ChR2(H134R)/EYFP) (*39*, *40*) and selecting offspring carrying both the CreETR and EYFP transgenes. The FosTRAP transgenic line Fos-iCre expresses iCre from an inducible c*-fos* promoter. IP injection of the tamoxifen metabolite, 4-OHT, allows iCre to translocate into the nucleus. Ai32 transgenic mice express channelrhodopsin-2/EYFP (ChR2-EYFP) following exposure to Cre recombinase. Crossing TRAP mice with Ai32 mice makes it possible to produce a stable transgenic line containing both Fos-iCre and ChR2-EYFP. All mice were socially housed in numbers 2 to 5 littermates, on a 12-hour light/dark cycle with access to food and water ad libitum. The day of birth was designated postnatal day 0 (P0). Infant (P17) mice were housed with parents until the time of weaning when they were group-housed by the same sex. Litter size was controlled, and no litter consisted of more than six pups. For infant experiments, each litter was counterbalanced across groups to limit litter effects. Each experimental group consisted of three to five liters on average. For MIA experiments, refer to table S1 for the exact number of litters per experimental group. All mice were 7 weeks old before undergoing surgery. After the operation, mice were allowed to recover for a minimum of 2 weeks in their home cage before experimentation. All procedures relating to mouse care and treatment conformed with the Health Products Regulatory Authority (HPRA) Ireland guidelines.

### ChR2-EYFP expression in Ai32-FosTRAP mice

To label memory engram cells with this system, mice were injected intraperitoneally with 4-OHT (50 mg/kg) 2 hours after the learning event. The Fos-iCre line expresses the inducible c*-fos* promoter. Injection of 4-OHT activates iCre recombinase. Activated iCre recombinase translocates to the nucleus and acts on two lox sites removing the stop codon that otherwise prevents the expression of the ChR2/EYFP transgene. ChR2/EYFP transgene expression is driven by the pCAG promoter in iCre-expressing tissue 72 hours after 4-OHT injection. For activity-dependent expression of ChR2-EYFP in Ai32-FosTRAP mice, kainic acid (5 mg/kg) was injected intraperitoneally followed by 4-OHT injection 2 hours after kainic acid injection.

### Stereotactic optical fiber implant

Mice were anesthetized using avertin (500 mg kg^−1^). Each animal underwent bilateral craniotomies using a 0.5-mm-diameter drill bit at −2.0 mm anteroposterior (AP) and ± 1.3 mm mediolateral (ML) for DG injections. A bilateral patch cord optical fiber implant (200-μm core diameter) was lowered above the injection site [−1.9-mm dorsoventral (DV)]. A layer of adhesive cement was applied to secure the optical fiber implant to the skull. Dental cement was applied to secure a protective cap to the implant and close the surgical site. Each animal received meloxicam analgesic (0.075 ml/5 g) via subcutaneous (SC) injection and remained on a heating pad until fully recovered from anesthesia. All mice were allowed to recover for 2 weeks before any subsequent experiments. All fiber sites were verified histologically.

### Immunohistochemistry

Mice were dispatched by overdose with 50 μl of sodium pentobarbital and perfused transcardially with PBS, followed by 4% paraformaldehyde (PFA) in PBS. Extracted brains were kept in 4% PFA at 4°C overnight and stored in PBS. Fifty-micrometer coronal slices were cut using a vibratome and collected in PBS. Slices were washed in PBS containing 0.2% Triton X-100 (PBS-T) followed by a 1-hour blocking in PBS-T with 10% normal goat serum at room temperature before being incubated with the primary antibody at 4°C overnight. Slices were washed in PBS-T 0.1% followed by a 1-hour incubation with the secondary antibody before undergoing another round of washing using PBS-T 0.1%. VECTASHIELD DAPI (4′,6-diamidino-2-phenylindole) was used to mount the slices onto super frost slides before visualization using an Olympus BX51 upright microscope. For cell counting experiments and dendritic spine analysis, an additional incubation with DAPI in PBS (1:1000) was carried out to label nuclei, and images were acquired using a Leica SP8 gated STED (stimulated emission depletion) nanoscope. All images were taken at ×40 magnification.

### Cell counting

To measure the extent to which populations of active cells overlap between the exposure to the same or different contexts, the number of EYFP^+^ and c-Fos^+^ immunoreactive neurons in the DG, AMG, RSC, and PAG was counted. All animals were euthanized 45 min after assay or optical stimulation for immunohistochemical analyses. Four coronal slices were taken from the dorsal hippocampus of each animal. For optogenetic stimulation experiments, only slices with accurate implant location sites were used for counting. Sections were imaged on a Leica SP8 gated STED nanoscope at a magnification of ×40. The area of interest was manually identified and the area of each region of interest was calculated using Fiji. To calculate the total number of DAPI cells in the DG, the average diameter of a sample of EYFP-positive cells in the DG was taken from each animal, and the area of the cell was calculated. The total number of cells was estimated as the area of the DG divided by the area of the cell. For the AMG, PAG, and RSC, the number of DAPI cells in three randomly selected regions of interest was counted and used along with the total area of the region to determine the total number of DAPI cells. The total number of c-Fos–positive and EYFP-positive cells were manually identified and counted for each region using Adobe Photoshop CC 2018. To calculate the percentage of cells expressing EYFP in each region, the total number of EYFP-positive cells was divided by the total number of DAPI-positive cells for each area. To calculate the percentage of cells expressing c-Fos in each region, the total number of c-Fos positive cells was divided by the total number of DAPI-positive cells for each area. Non-engram cell activation was quantified by subtracting the total number of cells positive for both EYFP and c-Fos from the total number of c-Fos–positive cells. To quantify engram reactivation, the number of EYFP- and c-Fos–positive cells was quantified as a percentage of total EYFP- or DAPI-positive cells. The level of chance was calculated by multiplying the percentage of EYFP^+^ cells with the total percentage of c-Fos^+^ cells and dividing by 100. This allowed for the determination of the chance of engram reactivation relative to the total number of EYFP^+^- and c-Fos^+^–labeled cells.

### Dendritic spine analysis

The dendrites of DG engram cells in Ai32-FosTRAP mice were analyzed after recall at P25. Dendritic spines were imaged using the Leica SP8 gated STED scope. *Z*-Stack images (10 μm) of 50-μm coronal brain sections were taken using Leica Application Suite X (LAS X) software (line average, 2; zoom factor, 1.7 to 1.9) at ×40. Imaris software (Oxford Instruments, Imaris v9.5) was used to carry out dendritic spine analysis. Dendrites were traced (10 μm) using a semiautomated neurofilament tracer tool and dendritic spines were individually highlighted and manually traced with the software. Each fragment represents a different ChR2-EYFP^+^ cell. Parameters were generated automatically using Imaris software. For analysis of dendritic spine head diameter and volume, an average for each fragment was taken and plotted.

### Behavioral assays

All behavioral experiments were conducted during the light cycle of the day (7:00 a.m. to 7:00 p.m.). All behavioral subjects were individually habituated to handling by the investigator for 3 min on 3 consecutive days. Handling took place in the breeding room. Immediately before each handling session, mice were transported in separate cages to and from the vicinity of the experimental room to habituate them to the journey.

### Contextual fear conditioning

Three distinct contexts were used for CFC. Context A was a 31 cm × 24 cm × 21 cm Med Associates chamber with a removable grid floor (bars, 3.2-mm diameter; space, 7.9 mm apart), opaque triangular ceiling, and scented with 0.25% benzaldehyde. Context B chambers were 29 cm × 25 cm × 22 cm Coulbourne Instruments chambers with Perspex white floors, bright white lighting, and scented with 1% acetic acid. All conditioning sessions were conducted in context A. Each session was 330 s in duration with three 0.75-mA shocks of 2-s duration delivered at 150, 210, and 270 s. Mice were removed from the conditioning chamber 60 s following the final footshock and returned to their home cage. All recall or context generalization tests were 180 s in duration. Testing conditions were identical to training conditioning except that no shocks were presented. Up to four mice were run simultaneously in the four identical chambers. Floors of chambers were cleaned with TriGene before and between runs. Mice were transported to and from the experimental room in separate Perspex cages. All experimental groups were counter-balanced for chambers within contexts.

### Contextual pre-exposure

For context pre-exposure, mice were exposed to context A (31 cm × 24 cm × 21 cm Med Associates chamber with a removable grid floor) for 10 min. Mice were removed from the chamber and placed back in their home cage. The following day, mice were placed back in context A where an immediate foot shock (1 mA, 2 s) was delivered. Mice were removed from the chamber 1 min after the shock and placed back in their home cage. Up to four mice were run simultaneously in the four identical chambers except for during the update when one mouse was run at a time. All recall or context specificity tests were 180 s in duration. Floors of chambers were cleaned with TriGene before and between runs.

### Novel object recognition

Each subject was allowed to habituate to the rectangular testing arena for 10 min on the day before object acquisition. During object acquisition, two identical objects were positioned on adjacent walls of the arena. Mice were allowed to explore both objects freely for a 10-min period before being placed back in their home cage. On testing, one familiar object was replaced with a novel object and each subject was introduced for a 5-min exploration period. Object exploration was defined as the time when the subject’s nose came within a 2-cm radius of the object. In between each trial, the testing arena and objects were cleaned with TriGene. For engram labeling experiments, P17 mice received a 4-OHT injection 2 hours after object acquisition. For optogenetic reactivation experiments, animals received 3 min of blue light stimulation directly before being placed into the testing arena.

### Barnes maze

Training consisted of five trials with a 10-min limit per trial. At the start of each trial, the mouse was placed in a black polyvinyl chloride start chamber located in the center of the maze apparatus. After 15 s, the start chamber was removed, and the latency and distance traveled to enter the escape tunnel were recorded. Subjects were placed back in their home cage and allowed 40 min in between each trial. The position of the escape tunnel remained in a fixed location relative to spatial cues in the room. For each probe trial, the escape tunnel was removed from the apparatus. Each mouse was allowed 5 min to freely explore the four quadrants of the maze before being removed and placed back in its home cage. The latency and distance traveled before reaching the location of the removed escape tunnel along with the time spent in each quadrant were recorded. The apparatus and escape tunnel were cleaned with TriGene after each trial. For engram labeling experiments, P17 mice received a 4-OHT injection 2 hours after the fifth trial. For optogenetic reactivation experiments, animals received 3 min of blue light stimulation directly before being placed into the testing arena.

### Three-chamber social interaction

Mice were habituated to the chamber with two empty holders for 10 min. The next day, mice were placed in the middle of the chamber and allowed to explore the three chambers for a period of 5 min. During this testing period, a social object (novel mouse) was contained in one holder in one chamber and an inanimate object (lego blocks) was contained in a holder in the other chamber. Object exploration and distance traveled were tracked using ANY-maze video tracking software.

### Repetition assay

A large home cage was filled 5 cm deep with wood chipping bedding and lightly tamped down to make an even flat surface. A consistent pattern of 20 identical glass marbles (15-mm diameter) was evenly placed (4 cm apart) on the surface of the wood chip bedding. Mice were left alone in the testing arena for 30 min. A picture was taken before and after the test for analysis. A marble was considered buried if two-thirds of the depth of the marble was buried.

### Optogenetic engram reactivation

Optogenetic engram reactivation was carried out in context C, a 31 cm × 24 cm × 21 cm Med Associates chamber with a white Perspex floor and curved insert, a dim light level, and scented with 0.25% acetophenone. The optical fiber implant was connected to a 45-nm laser diode fiber light source (Doric LDFLS 450/080). Habituation sessions lasted for 12 min. During testing, the 12-min session was divided into four 3-min epochs split into two light-off and two light-on epochs. During the light-on epochs, blue light stimulation (20 Hz) with a pulse width of 15 ms was delivered through the optical fiber implant for the entire 3-min duration. At the end of the 12-min session, the mouse was immediately detached from the laser and returned to its home cage.

### Artificial updating

Context A was a 31 × 24 × 21 cm Med Associates chamber with a removable Perspex black floor, black triangular ceiling, and scented with 0.25% benzaldehyde. Context B exposure was carried out in a rectangular testing arena. Artificial updating of a contextual memory was carried out in Context C, a 29 × 25 × 22 cm Coulbourne Instruments chamber with removable grid floors, bright white lighting, and scented with 1% acetic acid. Contexts A, B, and C were all located in different behavioral rooms. The optical fiber implant was connected to a 45 nm laser diode fiber light source (Doric LDFLS 450/080). The 7 min session consisted of a 2 min acclimatization period followed by 5 min of blue light stimulation (20 Hz) with a pulse width of 15 ms delivered through the optical fiber implant for the entire 5 min duration. Three shocks, 0.75 mA of 2 s duration, were delivered at minutes 4, 5, and 6 of the session. At the end of the 7 min session, the mouse was immediately detached from the laser and returned to its home cage.

### Maternal immune activation

Mice were mated overnight for one night. Females were weighed and checked for seminal plugs, noted as E0.5. Pregnant dams were injected subcutaneously with a single dose of poly(I:C) HMW (high molecular weight) (InvivoGen) at 20 mg/kg (*13*, *64*, *65*), rmlIL-17a (Immunotools) at 50 μg/kg, or control PBS on E12.5. For more detailed information, refer to table S2 (*66*). For rmlIL-17a injections at P17 or P25, mice were injected subcutaneously with a single dose of rmlIL-17a (Immunotools) at 50 ug/kg or control PBS 3 h before the recall test. For serum analysis by ELISA, blood serum was collected from female mice 24 h after subcutaneous administration of either PBS or poly(I:C) (20 mg/kg), and frozen at −20°C until further use. Blood serum was first diluted 1:5 and IL-17a levels were measured using the ELISA MAX Deluxe Set (Biolegend) as per the manufacturers’ instructions.

### Postnatal immune activation

Mice were injected subcutaneously with a single dose of poly(I:C) HMW (InvivoGen) at 20 mg/kg or control PBS on P3, P7, and P14.

### Genotyping

Genomic DNA was extracted from the ear punches of each mouse and sent to Transnetyx for genotyping.

### Statistical analysis

All experiments were analyzed blind to the experimental group. All videos were randomized before manual scoring. Behavioral performance was recorded by a digital video camera. Videos were manually scored individually, and investigators were blind to the experimental condition and test day during all manual scoring. Data analysis and statistics were conducted using GraphPad Prism 6.00 (GraphPad software) or RStudio v2023.03.0+386. Unpaired Student’s *t* tests were used for independent group comparisons. Paired Student’s *t* tests were used to assess differences within groups. Analysis of variance (ANOVA) followed by a Bonferroni post hoc test was used to determine conditions that were significant from each other where appropriate. Outliers were detected using Grubbs’ test where *P* < 0.05. For cell counting analysis, results were analyzed per mouse using nested *t* test or nested ANOVA followed by Bonferroni post hoc test, where each symbol represents *N* = 1. For MIA behavioral analysis, results were analyzed per litter (table S1) using nested *t* test or nested ANOVA followed by Bonferroni post hoc test. Pearson’s correlation coefficient was used to measure the relationship between two variables. Results are displayed as mean with SEM and deemed significant when *P* < 0.05.

## References

[1] C. Rovee-Collier, The development of infant memory. Curr. Dir. Psychol. Sci. 8, 80–85 (1999).

[2] N. S. Newcombe, A. B. Drummey, N. A. Fox, E. Lie, W. Ottinger-Alberts, Remembering early childhood: How much, how, and why (or why not). Curr. Dir. Psychol. Sci. 9, 55–58 (2000).

[3] B. A. Campbell, E. H. Campbell, Retention and extinction of learned fear in infant and adult rats. J. Comp. Physiol. Psychol. 55, 1–8 (1962). 13876002 10.1037/h0049182

[4] K. G. Akers, A. Martinez-Canabal, L. Restivo, A. P. Yiu, A. De Cristofaro, H.-L. L. Hsiang, A. L. Wheeler, A. Guskjolen, Y. Niibori, H. Shoji, K. Ohira, B. A. Richards, T. Miyakawa, S. A. Josselyn, P. W. Frankland, Hippocampal neurogenesis regulates forgetting during adulthood and infancy. Science 344, 598–602 (2014). 24812394 10.1126/science.1248903

[5] C. M. Alberini, A. Travaglia, Infantile amnesia: A critical period of learning to learn and remember. J. Neurosci. 37, 5783–5795 (2017). 28615475 10.1523/JNEUROSCI.0324-17.2017 PMC5473198

[6] B. L. Callaghan, R. Richardson, The effect of adverse rearing environments on persistent memories in young rats: Removing the brakes on infant fear memories. Transl. Psychiatry 2, e138 (2012). 22781171 10.1038/tp.2012.65 PMC3410617

[7] X. Liu, S. Ramirez, P. T. Pang, C. B. Puryear, A. Govindarajan, K. Deisseroth, S. Tonegawa, Optogenetic stimulation of a hippocampal engram activates fear memory recall. Nature 484, 381–385 (2012). 22441246 10.1038/nature11028 PMC3331914

[8] T. J. Ryan, D. S. Roy, M. Pignatelli, A. Arons, S. Tonegawa, Engram cells retain memory under retrograde amnesia. Science 348, 1007–1013 (2015). 26023136 10.1126/science.aaa5542 PMC5583719

[9] D. S. Roy, A. Arons, T. I. Mitchell, M. Pignatelli, T. J. Ryan, S. Tonegawa, Memory retrieval by activating engram cells in mouse models of early Alzheimer’s disease. Nature 531, 508–512 (2016). 26982728 10.1038/nature17172 PMC4847731

[10] J. N. Perusini, S. A. Cajigas, O. Cohensedgh, S. C. Lim, I. P. Pavlova, Z. R. Donaldson, C. A. Denny, Optogenetic stimulation of dentate gyrus engrams restores memory in Alzheimer’s disease mice. Hippocampus 27, 1110–1122 (2017). 28667669 10.1002/hipo.22756 PMC5610644

[11] K. Abdou, M. Shehata, K. Choko, H. Nishizono, M. Matsuo, S.-I. Muramatsu, K. Inokuchi, Synapse–specific representation of the identity of over–lapping memory engrams. Science 360, 1227–1231 (2018). 29903972 10.1126/science.aat3810

[12] T. J. Ryan, P. W. Frankland, Forgetting as a form of adaptive engram cell plasticity. Nat. Rev. Neurosci. 23, 173–186 (2022). 35027710 10.1038/s41583-021-00548-3

[13] G. B. Choi, Y. S. Yim, H. Wong, S. Kim, H. Kim, S. V. Kim, C. A. Hoeffer, D. R. Littman, J. R. Huh, The maternal interleukin–17a pathway in mice promotes autismlike phenotypes in offspring. Science 351, 933–939 (2016). 26822608 10.1126/science.aad0314 PMC4782964

[14] C. S. M. Cowan, R. Richardson, Early–life stress leads to sex–dependent changes in pubertal timing in rats that are reversed by a probiotic formulation. Dev. Psychobiol. 61, 679–687 (2019). 30043520 10.1002/dev.21765

[15] C. S. M. Cowan, A. A. Stylianakis, R. Richardson, Early-life stress, microbiota, and brain development: Probiotics reverse the effects of maternal separation on neural circuits underpinning fear expression and extinction in infant rats. Dev. Cogn. Neurosci. 37, 100627 (2019). 30981894 10.1016/j.dcn.2019.100627 PMC6969299

[16] S. M. Ohline, W. C. Abraham, Environmental enrichment effects on synaptic and cellular physiology of hippocampal neurons. Neuropharmacology 145, 3–12 (2019). 29634984 10.1016/j.neuropharm.2018.04.007

[17] B. T. Kalish, E. Kim, B. Finander, E. E. Duffy, H. Kim, C. K. Gilman, Y. S. Yim, L. Tong, R. J. Kaufman, E. C. Griffith, G. B. Choi, M. E. Greenberg, J. R. Huh, Maternal immune activation in mice disrupts proteostasis in the fetal brain. Nat. Neurosci. 24, 204–213 (2021). 33361822 10.1038/s41593-020-00762-9 PMC7854524

[18] R. M. Sullivan, M. Opendak, Defining immediate effects of sensitive periods on infant neurobehavioral function. Curr. Opin. Behav. Sci. 36, 106–114 (2020). 33043102 10.1016/j.cobeha.2020.08.006 PMC7543993

[19] J. H. Kim, R. Richardson, Immediate post-reminder injection of gamma-amino butyric acid (GABA) agonist midazolam attenuates reactivation of forgotten fear in the infant rat. Behav. Neurosci. 121, 1328–1332 (2007). 18085885 10.1037/0735-7044.121.6.1328

[20] J. H. Kim, G. P. McNally, R. Richardson, Recovery of fear memories in rats: Role of gamma-amino butyric acid (GABA) in infantile amnesia. Behav. Neurosci. 120, 40–48 (2006). 16492115 10.1037/0735-7044.120.1.40

[21] H. H. Y. Tang, G. P. McNally, R. Richardson, The effects of FG7142 on two types of forgetting in 18-day-old rats. Behav. Neurosci. 121, 1421–1425 (2007). 18085896 10.1037/0735-7044.121.6.1421

[22] A. Travaglia, R. Bisaz, E. S. Sweet, R. D. Blitzer, C. M. Alberini, Infantile amnesia reflects a developmental critical period for hippocampal learning. Nat. Neurosci. 19, 1225–1233 (2016). 27428652 10.1038/nn.4348 PMC5003643

[23] M. D. Reed, Y. S. Yim, R. D. Wimmer, H. Kim, C. Ryu, G. M. Welch, M. Andina, H. O. King, A. Waisman, M. M. Halassa, J. R. Huh, G. B. Choi, IL-17a promotes sociability in mouse models of neurodevelopmental disorders. Nature 577, 249–253 (2020). 31853066 10.1038/s41586-019-1843-6 PMC8112727

[24] Y. Shin Yim, A. Park, J. Berrios, M. Lafourcade, L. M. Pascual, N. Soares, J. Yeon Kim, S. Kim, H. Kim, A. Waisman, D. R. Littman, I. R. Wickersham, M. T. Harnett, J. R. Huh, G. B. Choi, Reversing behavioural abnormalities in mice exposed to maternal inflammation. Nature 549, 482–487 (2017). 28902835 10.1038/nature23909 PMC5796433

[25] A. M. Tartaglione, A. Villani, M. A. Ajmone-Cat, L. Minghetti, L. Ricceri, V. Pazienza, R. De Simone, G. Calamandrei, Maternal immune activation induces autism-like changes in behavior, neuroinflammatory profile and gut microbiota in mouse offspring of both sexes. Transl. Psychiatry 12, 384 (2022). 36104346 10.1038/s41398-022-02149-9 PMC9474453

[26] V. X. Han, S. Patel, H. F. Jones, R. C. Dale, Maternal immune activation and neuroinflammation in human neurodevelopmental disorders. Nat. Rev. Neurol. 17, 564–579 (2021). 34341569 10.1038/s41582-021-00530-8

[27] C. J. Zeiss, Comparative milestones in rodent and human postnatal central nervous system development. Toxicol. Pathol. 49, 1368–1373 (2021). 34569375 10.1177/01926233211046933

[28] S. Dutta, P. Sengupta, Men and mice: Relating their ages. Life Sci. 152, 244–248 (2016). 26596563 10.1016/j.lfs.2015.10.025

[29] V. Brust, P. M. Schindler, L. Lewejohann, Lifetime development of behavioural phenotype in the house mouse (Mus musculus). Front. Zool. 12 (Suppl. 1), S17 (2015). 26816516 10.1186/1742-9994-12-S1-S17 PMC4722345

[30] B. D. Semple, K. Blomgren, K. Gimlin, D. M. Ferriero, L. J. Noble-Haeusslein, Brain development in rodents and humans: Identifying benchmarks of maturation and vulnerability to injury across species. Prog. Neurobiol. 106–107, 1–16 (2013). 10.1016/j.pneurobio.2013.04.001 PMC3737272 23583307

[31] A. Guskjolen, J. W. Kenney, J. de la Parra, B.-R. A. Yeung, S. A. Josselyn, P. W. Frankland, Recovery of “lost” infant memories in mice. Curr. Biol. 28, 2283–2290.e3 (2018). 29983316 10.1016/j.cub.2018.05.059

[32] R. M. Vlasova, A.-M. Iosif, A. M. Ryan, L. H. Funk, T. Murai, S. Chen, T. A. Lesh, D. J. Rowland, J. Bennett, C. E. Hogrefe, R. J. Maddock, M. J. Gandal, D. H. Geschwind, C. M. Schumann, J. Van de Water, A. K. McAllister, C. S. Carter, M. A. Styner, D. G. Amaral, M. D. Bauman, Maternal immune activation during pregnancy alters postnatal brain growth and cognitive development in nonhuman primate offspring. J. Neurosci. 41, 9971–9987 (2021). 34607967 10.1523/JNEUROSCI.0378-21.2021 PMC8638691

[33] L. Shi, S. H. Fatemi, R. W. Sidwell, P. H. Patterson, Maternal influenza infection causes marked behavioral and pharmacological changes in the offspring. J. Neurosci. 23, 297–302 (2003). 12514227 10.1523/JNEUROSCI.23-01-00297.2003 PMC6742135

[34] M. L. Estes, A. K. McAllister, Maternal immune activation: Implications for neuropsychiatric disorders. Science 353, 772–777 (2016). 27540164 10.1126/science.aag3194 PMC5650490

[35] S. E. P. Smith, J. Li, K. Garbett, K. Mirnics, P. H. Patterson, Maternal immune activation alters fetal brain development through interleukin-6. J. Neurosci. 27, 10695–10702 (2007). 17913903 10.1523/JNEUROSCI.2178-07.2007 PMC2387067

[36] A. Douglas, B. Stevens, L. Lynch, Interleukin-17 as a key player in neuroimmunometabolism. Nat. Metab. 5, 1088–1100 (2023). 37488456 10.1038/s42255-023-00846-3 PMC10440016

[37] C. J. Guenthner, K. Miyamichi, H. H. Yang, H. C. Heller, L. Luo, Permanent genetic access to transiently active neurons via TRAP: Targeted recombination in active populations. Neuron 78, 773–784 (2013). 23764283 10.1016/j.neuron.2013.03.025 PMC3782391

[38] W. E. Allen, L. A. DeNardo, M. Z. Chen, C. D. Liu, K. M. Loh, L. E. Fenno, C. Ramakrishnan, K. Deisseroth, L. Luo, Thirst-associated preoptic neurons encode an aversive motivational drive. Science 357, 1149–1155 (2017). 28912243 10.1126/science.aan6747 PMC5723384

[39] L. Madisen, T. A. Zwingman, S. M. Sunkin, S. W. Oh, H. A. Zariwala, H. Gu, L. L. Ng, R. D. Palmiter, M. J. Hawrylycz, A. R. Jones, E. S. Lein, H. Zeng, A robust and high-throughput Cre reporting and characterization system for the whole mouse brain. Nat. Neurosci. 13, 133–140 (2010). 20023653 10.1038/nn.2467 PMC2840225

[40] L. Madisen, T. Mao, H. Koch, J. Zhuo, A. Berenyi, S. Fujisawa, Y.-W. A. Hsu, A. J. Garcia, X. Gu, S. Zanella, J. Kidney, H. Gu, Y. Mao, B. M. Hooks, E. S. Boyden, G. Buzsáki, J. M. Ramirez, A. R. Jones, K. Svoboda, X. Han, E. E. Turner, H. Zeng, A toolbox of Cre-dependent optogenetic transgenic mice for light-induced activation and silencing. Nat. Neurosci. 15, 793–802 (2012). 22446880 10.1038/nn.3078 PMC3337962

[41] J. Li, R. Y. Jiang, K. L. Arendt, Y.-T. Hsu, S. R. Zhai, L. Chen, Defective memory engram reactivation underlies impaired fear memory recall in Fragile X syndrome. eLife 9, e61882 (2020). 33215988 10.7554/eLife.61882 PMC7679137

[42] P. Coiro, R. Padmashri, A. Suresh, E. Spartz, G. Pendyala, S. Chou, Y. Jung, B. Meays, S. Roy, N. Gautam, Y. Alnouti, M. Li, A. Dunaevsky, Impaired synaptic development in a maternal immune activation mouse model of neurodevelopmental disorders. Brain Behav. Immun. 50, 249–258 (2015). 26218293 10.1016/j.bbi.2015.07.022 PMC4955953

[43] S. Ramirez, X. Liu, P.-A. Lin, J. Suh, M. Pignatelli, R. L. Redondo, T. J. Ryan, S. Tonegawa, Creating a false memory in the hippocampus. Science 341, 387–391 (2013). 23888038 10.1126/science.1239073

[44] K. Z. Tanaka, H. He, A. Tomar, K. Niisato, A. J. Y. Huang, T. J. McHugh, The hippocampal engram maps experience but not place. Science 361, 392–397 (2018). 30049878 10.1126/science.aat5397

[45] R. L. Redondo, J. Kim, A. L. Arons, S. Ramirez, X. Liu, S. Tonegawa, Bidirectional switch of the valence associated with a hippocampal contextual memory engram. Nature 513, 426–430 (2014). 25162525 10.1038/nature13725 PMC4169316

[46] M. L. Reger, D. A. Hovda, C. C. Giza, Ontogeny of rat recognition memory measured by the novel object recognition task. Dev. Psychobiol. 51, 672–678 (2009). 19739136 10.1002/dev.20402 PMC2956740

[47] B. Bessières, M. Jia, A. Travaglia, C. M. Alberini, Developmental changes in plasticity, synaptic, glia, and connectivity protein levels in rat basolateral amygdala. Learn. Mem. 26, 436–448 (2019). 31615855 10.1101/lm.049866.119 PMC6796789

[48] G. Haley, J. Raber, Spatial learning and memory in animal models and humans, in *Animal Models of Behavioral Analysis* (Humana Press, 2010), vol. 50, pp. 91–109.

[49] A. Garthe, J. Behr, G. Kempermann, Adult-generated hippocampal neurons allow the flexible use of spatially precise learning strategies. PLOS ONE 4, e5464 (2009). 19421325 10.1371/journal.pone.0005464 PMC2674212

[50] A. Garthe, I. Roeder, G. Kempermann, Mice in an enriched environment learn more flexibly because of adult hippocampal neurogenesis. Hippocampus 26, 261–271 (2016). 26311488 10.1002/hipo.22520 PMC5049654

[51] G. Berdugo-Vega, G. Arias-Gil, A. López-Fernández, B. Artegiani, J. M. Wasielewska, C.-C. Lee, M. T. Lippert, G. Kempermann, K. Takagaki, F. Calegari, Increasing neurogenesis refines hippocampal activity rejuvenating navigational learning strategies and contextual memory throughout life. Nat. Commun. 11, 135 (2020). 31919362 10.1038/s41467-019-14026-z PMC6952376

[52] M. F. López-Aranda, I. Chattopadhyay, G. M. Boxx, E. R. Fraley, T. K. Silva, M. Zhou, M. Phan, I. Herrera, S. Taloma, R. Mandanas, K. Bach, M. Gandal, D. H. Geschwind, G. Cheng, A. Rzhetsky, S. A. White, A. J. Silva, Postnatal immune activation causes social deficits in a mouse model of tuberous sclerosis: Role of microglia and clinical implications. Sci Adv 7, eabf2073 (2021). 34533985 10.1126/sciadv.abf2073 PMC8448451

[53] S. Poll, M. Mittag, F. Musacchio, L. C. Justus, E. A. Giovannetti, J. Steffen, J. Wagner, L. Zohren, S. Schoch, B. Schmidt, W. S. Jackson, D. Ehninger, M. Fuhrmann, Memory trace interference impairs recall in a mouse model of Alzheimer’s disease. Nat. Neurosci. 23, 952–958 (2020). 32514139 10.1038/s41593-020-0652-4

[54] L. Autore, J. D. O’Leary, C. O. S. Luis, T. J. Ryan, Adaptive expression of engrams by retroactive interference. Cell Rep. 42, 11299 (2023). 10.1016/j.celrep.2023.112999 37590145

[55] S. Tonegawa, M. Pignatelli, D. S. Roy, T. J. Ryan, Memory engram storage and retrieval. Curr. Opin. Neurobiol. 35, 101–109 (2015). 26280931 10.1016/j.conb.2015.07.009

[56] D. S. Roy, Y.-G. Park, M. E. Kim, Y. Zhang, S. K. Ogawa, N. DiNapoli, X. Gu, J. H. Cho, H. Choi, L. Kamentsky, J. Martin, O. Mosto, T. Aida, K. Chung, S. Tonegawa, Brain-wide mapping reveals that engrams for a single memory are distributed across multiple brain regions. Nat. Commun. 13, 1799 (2022). 35379803 10.1038/s41467-022-29384-4 PMC8980018

[57] S. Ramirez, X. Liu, C. J. MacDonald, A. Moffa, J. Zhou, R. L. Redondo, S. Tonegawa, Activating positive memory engrams suppresses depression-like behaviour. Nature 522, 335–339 (2015). 26085274 10.1038/nature14514 PMC5583720

[58] T. J. Ryan, C. Ortega-de San Luis, M. Pezzoli, S. Sen, Engram cell connectivity: An evolving substrate for information storage. Curr. Opin. Neurobiol. 67, 215–225 (2021). 33812274 10.1016/j.conb.2021.01.006

[59] E. Atanasova, A. P. Arévalo, I. Graf, R. Zhang, J. Bockmann, A.-K. Lutz, T. M. Boeckers, Immune activation during pregnancy exacerbates ASD-related alterations in Shank3-deficient mice. Mol. Autism. 14, 1 (2023). 36604742 10.1186/s13229-022-00532-3 PMC9814193

[60] T. Dong, J. He, S. Wang, L. Wang, Y. Cheng, Y. Zhong, Inability to activate Rac1-dependent forgetting contributes to behavioral inflexibility in mutants of multiple autism-risk genes. Proc. Natl. Acad. Sci. U.S.A. 113, 7644–7649 (2016). 27335463 10.1073/pnas.1602152113 PMC4941477

[61] V. Zamoscik, D. Mier, S. N. L. Schmidt, P. Kirsch, Early memories of individuals on the autism spectrum assessed using online self-reports. Front. Psych. 7, 79 (2016). 10.3389/fpsyt.2016.00079 PMC4852178 27199786

[62] B. A. Campbell, J. R. Misanin, B. C. White, L. D. Lytle, Species differences in ontogeny of memory: Indirect support for neural maturation as a determinant of forgetting. J. Comp. Physiol. Psychol. 87, 193–202 (1974).

[63] L. L. Shook, E. L. Sullivan, J. O. Lo, R. H. Perlis, A. G. Edlow, COVID-19 in pregnancy: Implications for fetal brain development. Trends Mol. Med. 28, 319–330 (2022). 35277325 10.1016/j.molmed.2022.02.004 PMC8841149

[64] C. Cunningham, S. Campion, J. Teeling, L. Felton, V. H. Perry, The sickness behaviour and CNS inflammatory mediator profile induced by systemic challenge of mice with synthetic double-stranded RNA (poly I:C). Brain Behav. Immun. 21, 490–502 (2007). 17321719 10.1016/j.bbi.2006.12.007

[65] N. McGarry, C. L. Murray, S. Garvey, A. Wilkinson, L. Tortorelli, L. Ryan, L. Hayden, D. Healy, E. W. Griffin, E. Hennessy, M. Arumugam, D. T. Skelly, K. J. Mitchell, C. Cunningham, Double stranded RNA drives anti-viral innate immune responses, sickness behavior and cognitive dysfunction dependent on dsRNA length, IFNAR1 expression and age. Brain Behav. Immun. 95, 413–428 (2021). 33892139 10.1016/j.bbi.2021.04.016 PMC8447494

[66] A. C. Kentner, S. D. Bilbo, A. S. Brown, E. Y. Hsiao, A. K. McAllister, U. Meyer, B. D. Pearce, M. V. Pletnikov, R. H. Yolken, M. D. Bauman, Maternal immune activation: Reporting guidelines to improve the rigor, reproducibility, and transparency of the model. Neuropsychopharmacology 44, 245–258 (2019). 30188509 10.1038/s41386-018-0185-7 PMC6300528

