# Supplementary material for: Immune activation state modulates infant engram expression across development
Source: Sci Adv. 2023 Nov 8;9(45):eadg9921. doi: 10.1126/sciadv.adg9921 (PMC10631722; doi:10.1126/sciadv.adg9921)
Supplement: Supplementary file 1 — Figs. S1 to S16 Tables S1 and S2 [file sciadv.adg9921_sm.pdf]

Science Advances
AAAS

Supplementary Materials for

Immune activation state modulates infant engram expression across development

Sarah D. Power *et al.*

Corresponding author: Tomás J. Ryan, tomas.ryan@tcd.ie

Sci. Adv. **9**, eadg9921 (2023)
DOI: 10.1126/sciadv.adg9921

This PDF file includes:

Figs. S1 to S16
Tables S1 and S2

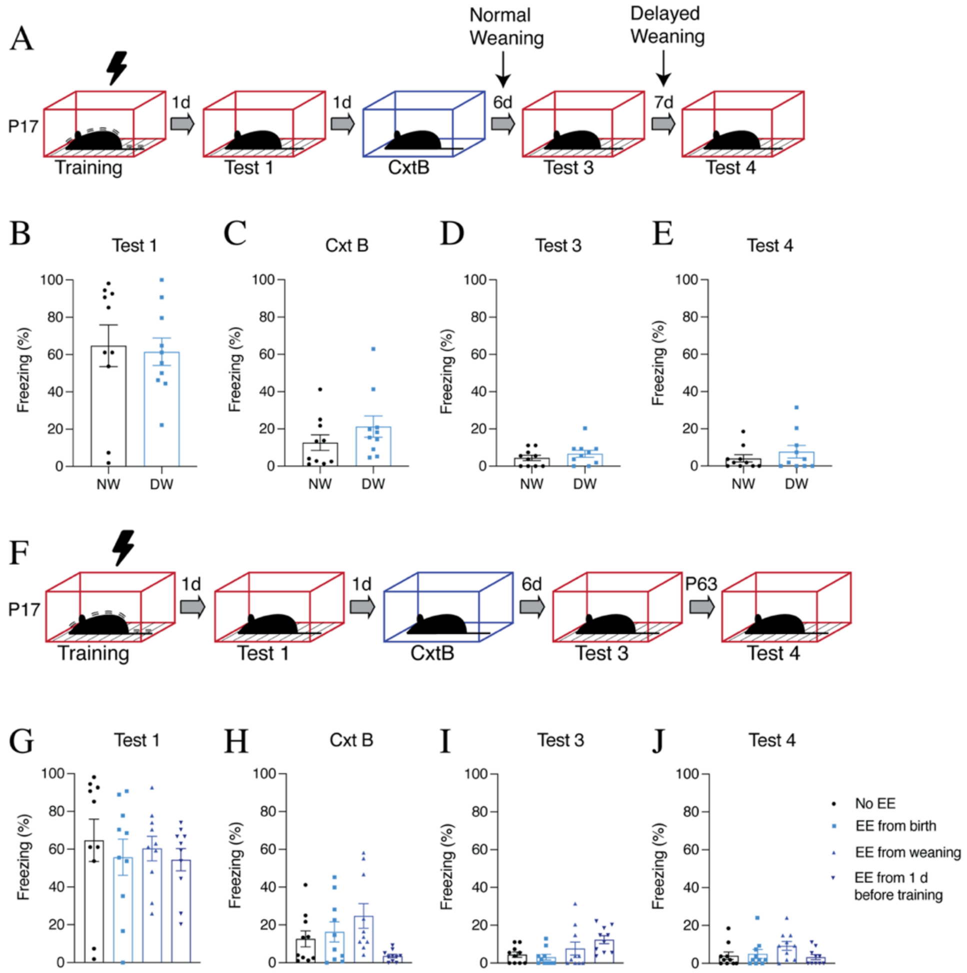

Fig. S1. Delayed weaning or environmental enrichment does not affect infantile amnesia.
(**A**) Behavioral schedule for CFC in infant (P17) C57BL/6J male mice subjected to normal (P21) or delayed (P24) weaning. The lightning symbol represents footshocks. (**B-E**) Freezing levels during recall tests (n=10) at Test 1 (P18), CxtB (P19), Test 3 (P25), and Test 4 (P32). (**F**) Behavioral schedule for CFC in infant C57BL/6J male mice. (**G-J**) Freezing levels during recall tests (n=10). $P < 0.05$, $P < 0.01$, $P < 0.001$ calculated by (**B-E)** Student t-test or (**G-J**) ANOVA with Bonferroni post hoc tests. Data presented as ±SEM.

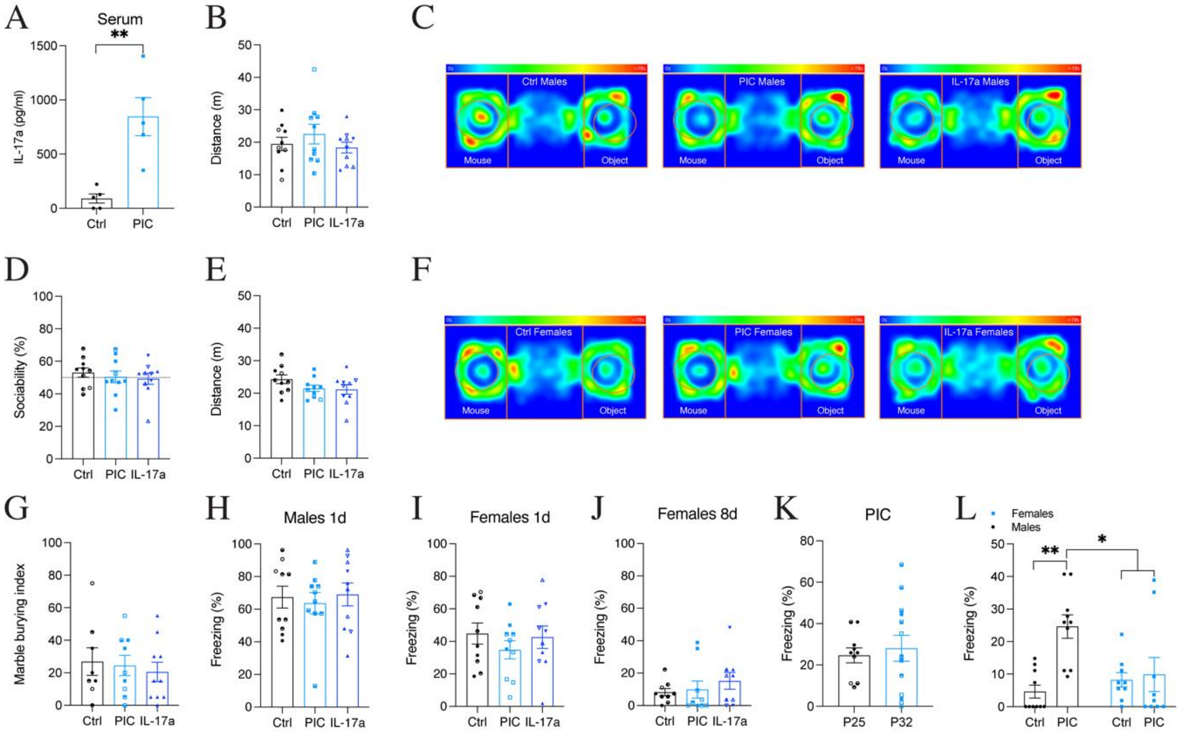

Fig. S2. MIA female offspring do not demonstrate social interaction deficits or repetitive behavior but do show infantile amnesia for a fear memory.

(**A**) IL-17a cytokine levels in blood serum of female C57 mice injected with PBS (Ctrl n=5) or Poly I:C (PIC n=5). (**B**, **E**) Total distance (m) traveled during social interaction test in adult (P63) C57BL/6J (**B**) male (n=10) and (**E**) female (n=10) MIA offspring. (**C**, **F**) Heat map analysis of time spent in each chamber during social interaction task. (**D**) Social interaction index (%) of adult (P63) C57BL/6J female MIA offspring (n=10). (**G**) Number of marbles buried by adult female MIA offspring during marble burying task (n= 8-10). (**H**) Memory recall for CFC in context A for male MIA offspring testing 1 d after training (n=10). (**I**, **J**) Recall for CFC in context A for female MIA offspring tested (**I**) 1 d (n=9-10) or (**J**) 8 d (n= 9) after training. (**K**) Memory recall for CFC in context A for male MIA offspring tested 8 d (P25) (n=10) or 15 d (P32) (n=13) after training. (**L**) Memory recall comparison in male and female MIA offspring 8 d after training. $P < 0.05$, $P < 0.01$, $P < 0.001$ calculated by (**A**) Student t-test, (**K**) nested Student t-test, (**B**, **D**, **E**, **G**-**J**) nested ANOVA, or (**L**) two-way ANOVA with Bonferroni post hoc tests. Data presented as ±SEM.

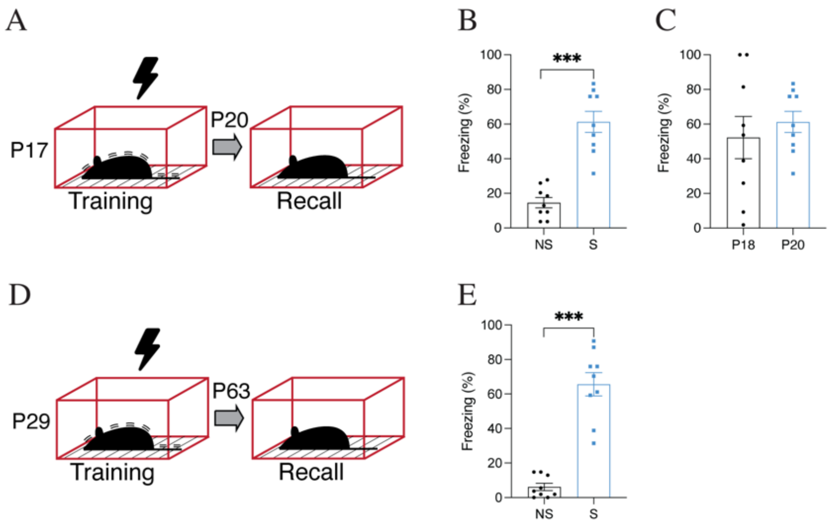

Fig. S3. Infant mice show similar memory retention when tested 1 or 3 d after training at P17. (**A**) Behavioral schedule for CFC in C57BL/6J P17 mice. The syringe symbol represents 4-OHT injection 2 h after exposure. The black lightning symbol represents foot shocks. (**B**, **C**) Freezing levels (%) during recall in context A. No shock (NS), shock (S). (**D**) Behavioral schedule for CFC in C57BL/6J P29 mice. (**E**) Freezing levels (%) during recall in context A at P63. $P < 0.05$, $P < 0.01$, $P < 0.001$ calculated by (**B**, **C**, **E)** Student t-test. Data presented as ±SEM.

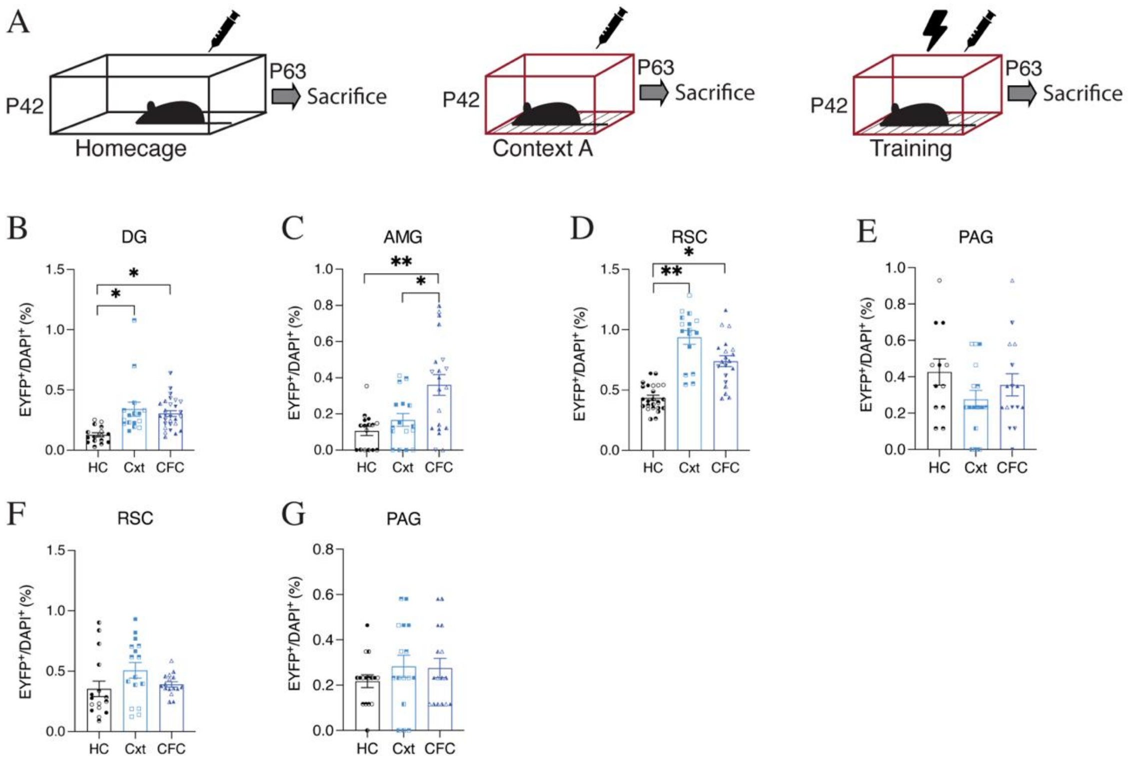

Fig. S4. Adult Ai32-FosTRAP mice show activity-dependent increases in ChR2-EYFP labeling. (**A**) Behavioral schedule. The syringe symbol represents 4-OHT injection 2 h after exposure. The black lightning symbol represents foot shocks. (**B**-**E**) Quantification of ChR2-EYFP$^+$ cells in the (**B**) DG (N=4-7, n=4), (**C**) AMG (N=4-5, n=4), (**D**) RSC (N=4-6, n=4) and (**E**) PAG (N=3-4, n=4) for an adult (P42) encoded homecage (HC), context (Cxt) and contextual fear (CFC) memory. Exposure to a context resulted in significantly more ChR2-EYFP$^+$ cells in the (**B**) DG ($p < 0.001$) and (**D**) RSC ($p < 0.001$). Quantification of ChR2-EYFP$^+$ cells after CFC was significantly higher in the (**B**) DG ($p < 0.05$), (**C**) AMG ($p < 0.01$), and the (**D**) RSC ($p < 0.05$) compared to HC control. (**F**, **G**) ChR2-EYFP$^+$ cell counts (N=4, n=4) in the RSC and PAG for an infant (P17) encoded HC, Cxt, and CFC memory. $P < 0.05$, $P < 0.01$, $P < 0.001$ calculated by (**B**-**G**) nested ANOVA with Bonferroni post hoc tests. Data presented as ±SEM.

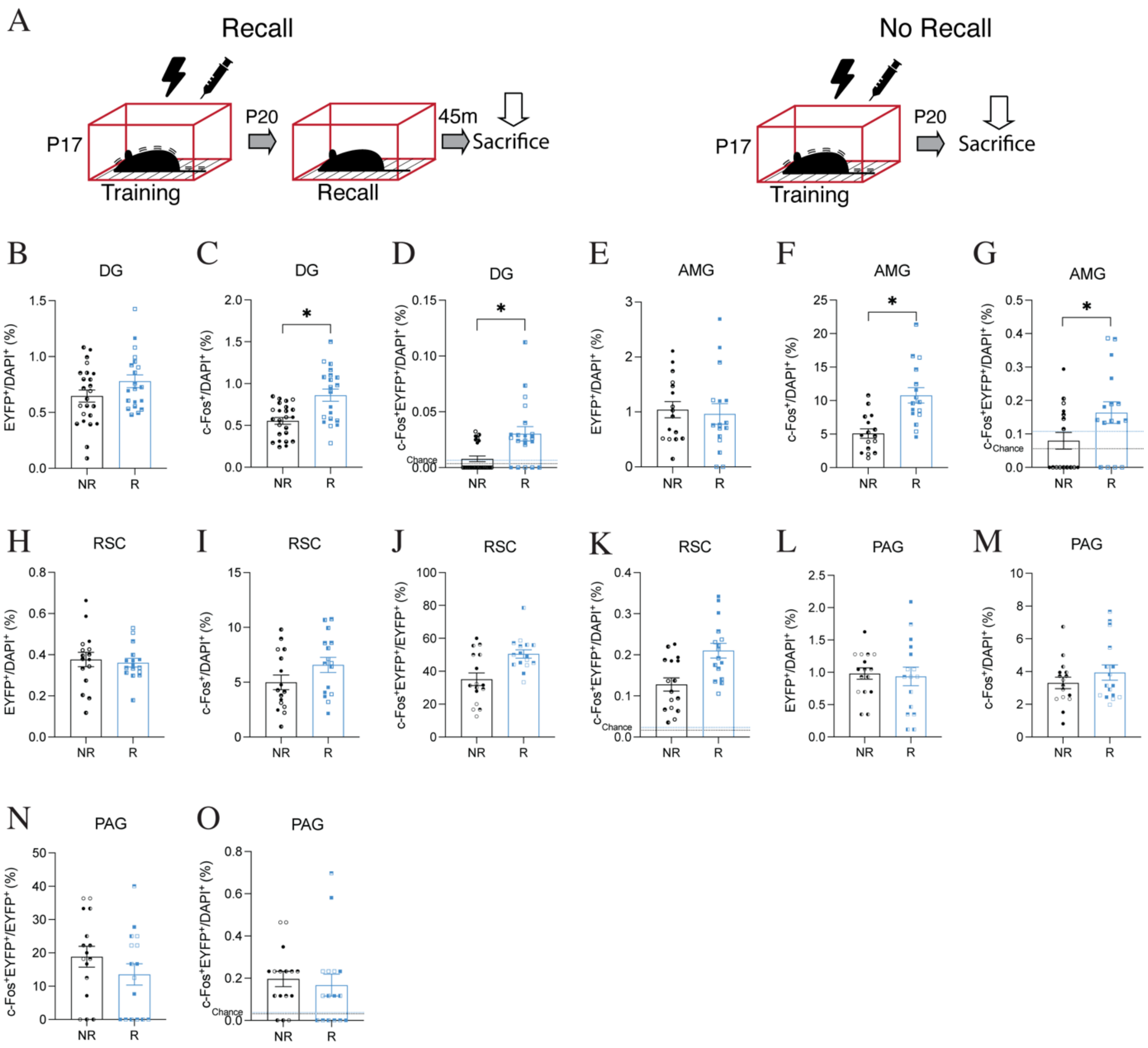

Fig. S5. Engram reactivation is increased in the DG and AMG after natural recall at P20.
(**A**) Behavioral paradigm. (**B-O**) Histological analysis of cell counts after recall at P20. Percentage of (**B**) ChR2-EYFP$^+$ and (**C**) c-Fos$^+$ cells in the DG (N=5-6, n=4). There was a significant increase in c-Fos$^+$ cells in the DG after recall at P20 ($p < 0.05$). (**D**) Percentage of DAPI cells positive for both ChR2-EYFP and c-Fos. (**D**) Engram reactivation as a percentage of DAPI$^+$ was significantly higher in the DG after recall at P20 ($p < 0.05$). Percentage of (**E**) ChR2-EYFP$^+$ and (**F**) c-Fos$^+$ cells in the AMG (N=4, n=4). There was a significant increase in c-Fos$^+$ cells in the AMG after recall at P20 ($p < 0.05$). (**G**) Percentage of DAPI cells positive for both ChR2-EYFP and c-Fos. (**G**) Engram reactivation as a percentage of DAPI$^+$ in the AMG was significantly higher after recall at P20 ($p < 0.05$). Percentage of (**H**) ChR2-EYFP$^+$ and (**I**) c-Fos$^+$ cells in the RSC (N=4, n=4). Engram reactivation in the RSC as a percentage of (**J**) ChR2-EYFP$^+$ and (**K**) DAPI$^+$ cells.

Percentage of (**L**) ChR2-EYFP^{+} and (**M**) c-Fos^{+} cells in the PAG (N=4, n=4). Engram reactivation in the PAG as a percentage of (**N**) ChR2-EYFP^{+} and (**O**) DAPI^{+} cells. $P < 0.05$, $P < 0.01$, $P < 0.001$ calculated by (**B**-**O**) nested t-test. Data presented as ±SEM.

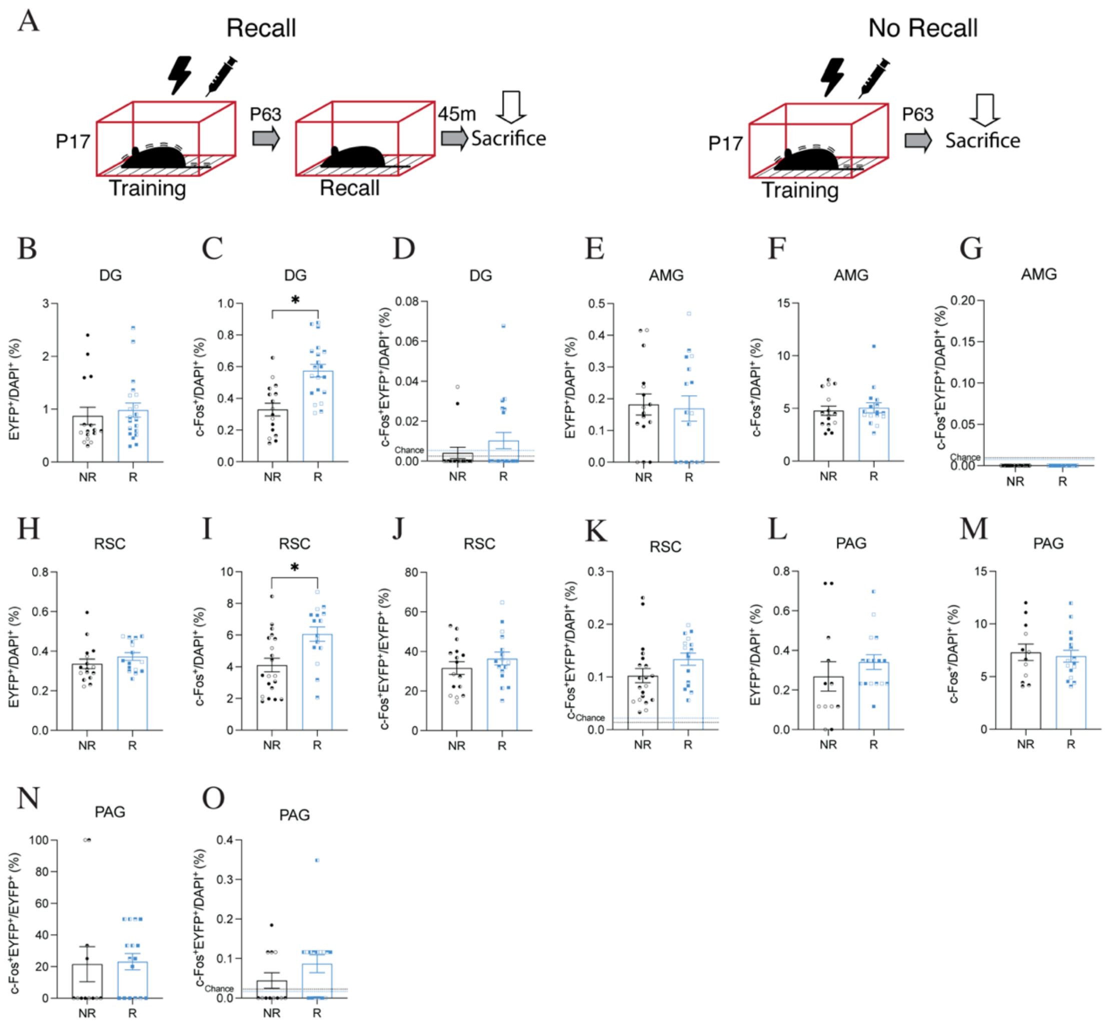

Fig. S6. Presentation of recall cues for an infant encoded context does not result in engram reactivation at P63.

(**A**) Behavioral paradigm. (**B**-**O**) Histological analysis of cell counts after recall at P63. Percentage of (**B**, **E**) ChR2-EYFP$^+$ and (**C**, **F**) c-Fos$^+$ cells in the DG (N=4-5, n=4) and AMG (N=4, n=4). (**C**) There was a significant increase in c-Fos$^+$ cells in the DG after recall at P63 ($p < 0.05$). (**D**, **G**) Engram reactivation as a percentage of DAPI$^+$ in the DG and AMG. Percentage of (**H**, **L**) ChR2-EYFP$^+$ and (**I**, **M**) c-Fos$^+$ cells in the RSC (N=4-5, n=4) and PAG (N=3-4, n=4). (**I**) There was a significant increase in c-Fos$^+$ cells in the RSC after recall at P63 ($p < 0.05$). Engram reactivation in the RSC and PAG as a percentage of (**J**, **N**) ChR2-EYFP$^+$ and (**K**, **O**) DAPI$^+$ cells. $P < 0.05$, $P < 0.01$, $P < 0.001$ calculated by (**B**-**O**) nested Student t-test. Data presented as ±SEM.

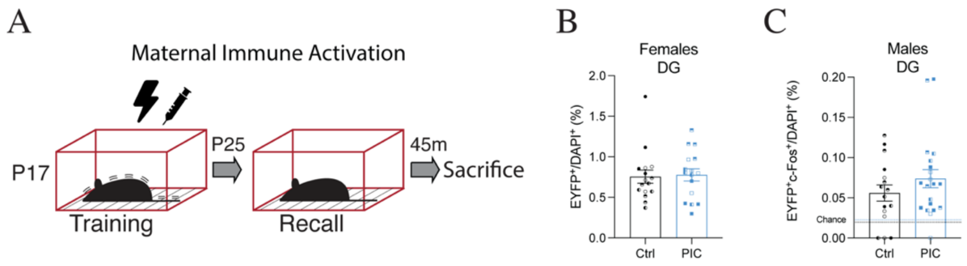

Fig. S7. MIA female offspring do not show an increased number of EYFP$^+$ cells in the DG.
(**A**) Behavioral paradigm for labeling engram cells in MIA female Ai32-FosTRAP offspring. (**B**) ChR2-EYFP$^+$ cell counts in the DG (N= 4, n=4) of female MIA offspring after recall at P25. (**C**) Engram reactivation in the DG of male MIA offspring as a percentage of DAPI$^+$ cells. $P < 0.05$, $P < 0.01$, $P < 0.001$ calculated by (**B**, **C**) nested Student t-test. Data presented as ±SEM.

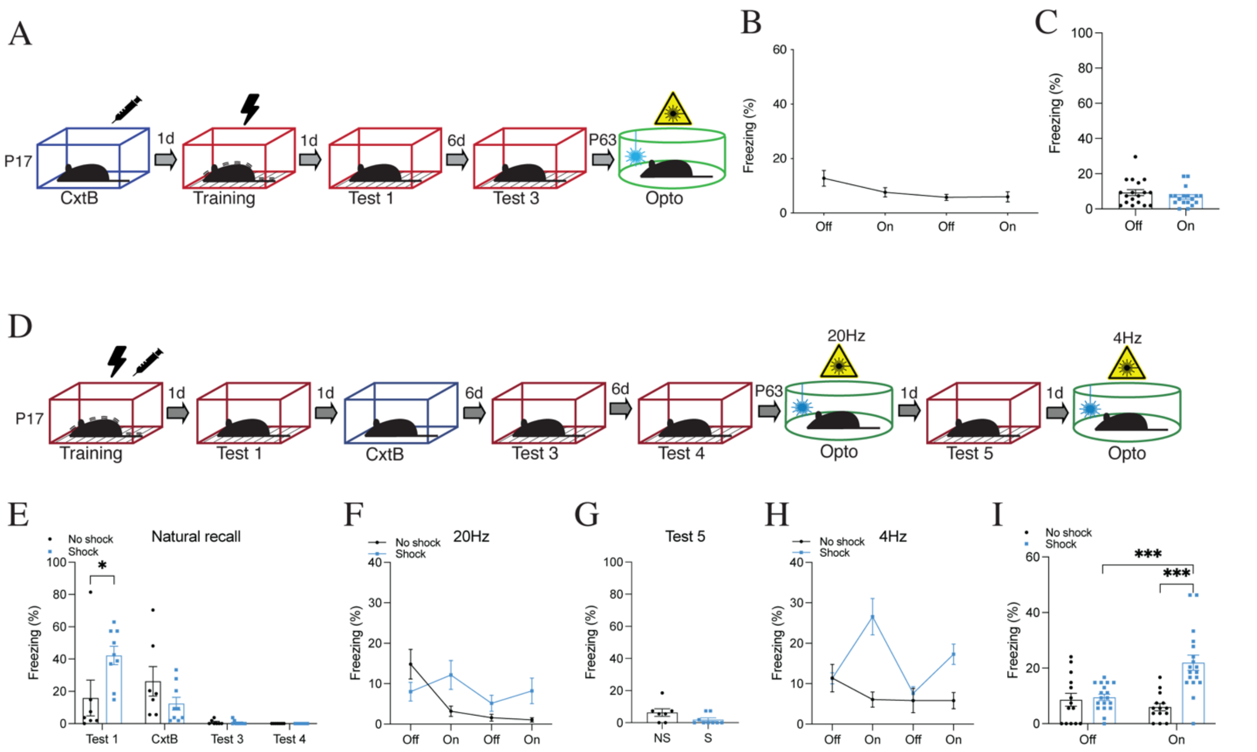

Fig. S8 Optogenetic reactivation of an infant engram in the CA1 elicits freezing behavior in adult mice.

(**A**) Behavioral schedule for engram reactivation of a neutral context B in the DG of Ai32-FosTRAP male mice. The black lightning symbol represents foot shocks. The syringe symbol represents 4-OHT injection 2 h after context exposure. (**B**) Memory recall in context C (engram reactivation) with light-off and light-on epochs (n=9). (**C**) Freezing for the two light-off and light-on epochs averaged. (**D**) Behavioural schedule for infant engram reactivation in the CA1 of adult Ai32-FosTRAP male mice (n=7) (NS), (n=9) (S). (**E**, **G**) Freezing levels during natural memory recall. (**F**, **H)** Memory recall in context C during engram reactivation at (**F**) 20 Hz or (**H**) 4 Hz with light-off and light-on epochs. (**I**) Freezing for the two light-off and light-on epochs during 4 Hz stimulation averaged. *$P < 0.05$, $P < 0.01$, $P < 0.001$* calculated by (**C**, **G**) Student t-test or (**E**, **I**) two-way ANOVA with Bonferroni post hoc tests. Data presented as ±SEM.

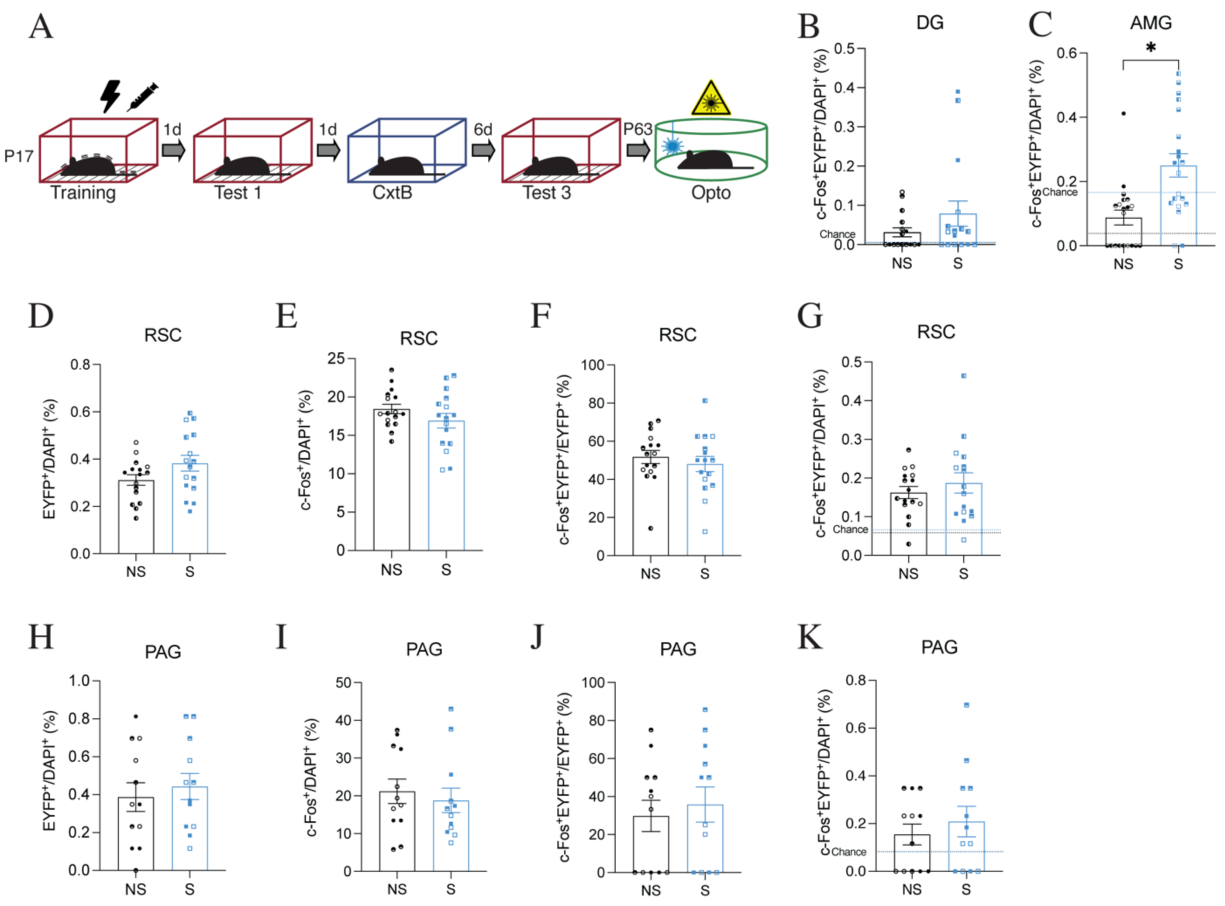

Fig. S9. Optogenetic reactivation of an infant encoded engram in adult mice.

(**A**) Behavioral paradigm. (**B-K**) Cell counts after optogenetic reactivation of an infant encoded engram in adult Ai32-FosTRAP mice. No shock (NS), shock (S). (**B**, **C**) Percentage of DAPI cells positive for both ChR2-EYFP and c-Fos in the DG and AMG. (**C**) Engram reactivation as a percentage of DAPI$^+$ cells was significantly higher ($p < 0.05$) in the AMG (N=5, n=4) after optogenetic stimulation of an infant-encoded fear memory. Percentage of (**D**) ChR2-EYFP$^+$ and (**E**) c-Fos$^+$ cells in the RSC (N=4, n=4). Engram reactivation in the RSC as a percentage of (**F**) ChR2-EYFP$^+$ and (**G**) DAPI$^+$ cells. Percentage of (**H**) ChR2-EYFP$^+$ and (**I**) c-Fos$^+$ cells in the PAG (N=4, n=3-4). Engram reactivation in the PAG as a percentage of (**J**) ChR2-EYFP$^+$ and (**K**) DAPI$^+$ cells. $P < 0.05$, $P < 0.01$, $P < 0.001$ calculated by (**B-K**) nested Student t-test. Data presented as ±SEM.

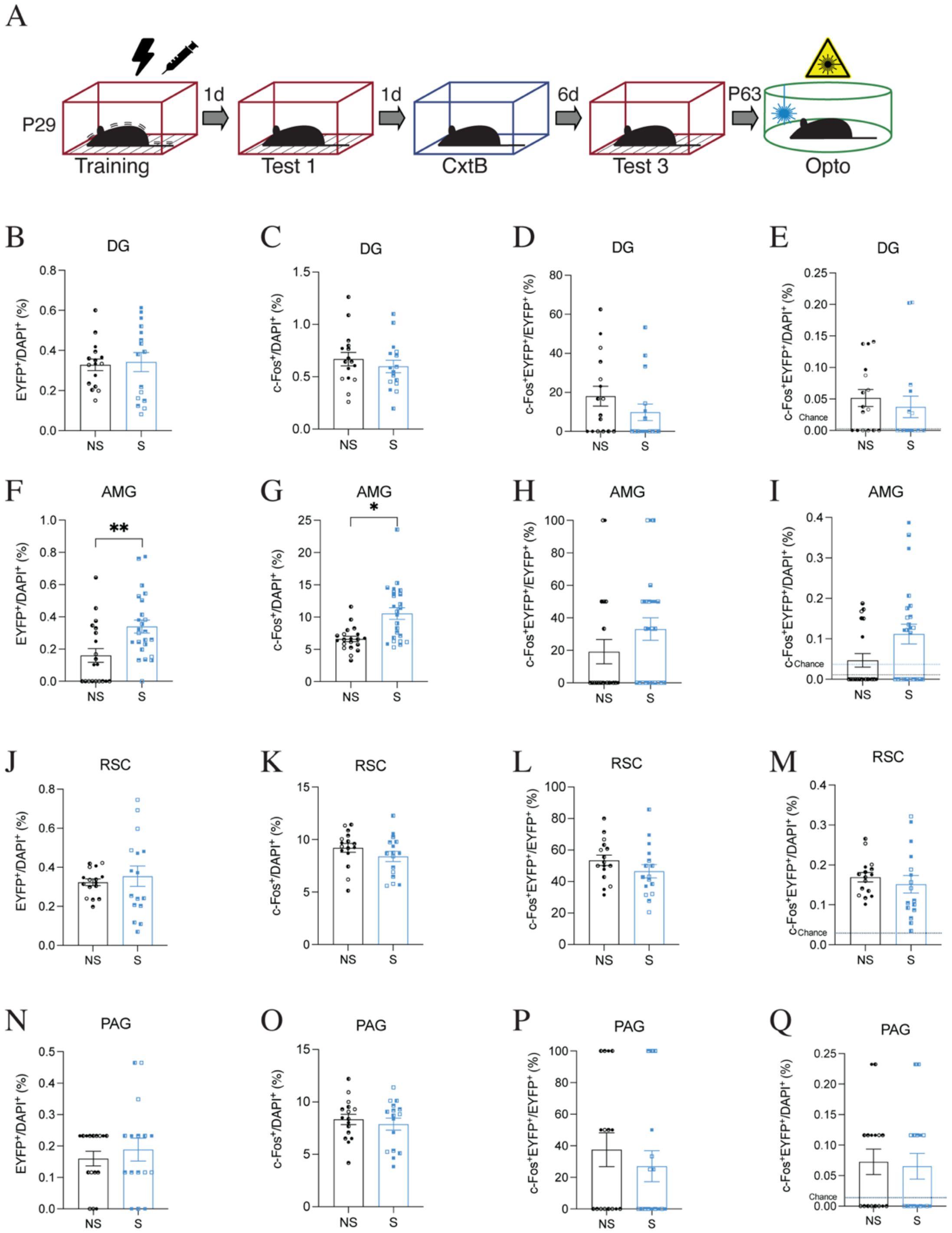

Fig. S10 Optogenetic stimulation of an CFC engram encoded after the infantile period causes an increase in activity in the amygdala in adult mice.

(**A**) Behavioral schedule for optogenetic reactivation of DG engram cells, labeled at P29, in adult (P63) Ai32-FosTRAP mice. The black lightning symbol represents foot shocks. The syringe symbol represents 4-OHT injection 2 h after training. Percentage of ChR2-EYFP^{+} cells labeled

during training in the (**B**) DG (N=4, n=4), (**F**) AMG (N=5-6, n=4) ($p < 0.05$), (**J**) RSC (N=4, n=4), and (**N**) PAG (N=4, n=4). Percentage of c-Fos^{+} cells in the (**C**) DG, (**G**) AMG, (**K**) RSC, and (**O**) PAG after optogenetic stimulation at P63. Engram reactivation as a percentage of (**D**, **H**, **L**, **P**) EYFP^{+} cells and (**E**, **I**, **M**, **Q**) DAPI^{+} cells. $P < 0.05$, $P < 0.01$, $P < 0.001$ calculated by (**B-Q**) nested Student t-test. Data presented as ±SEM.

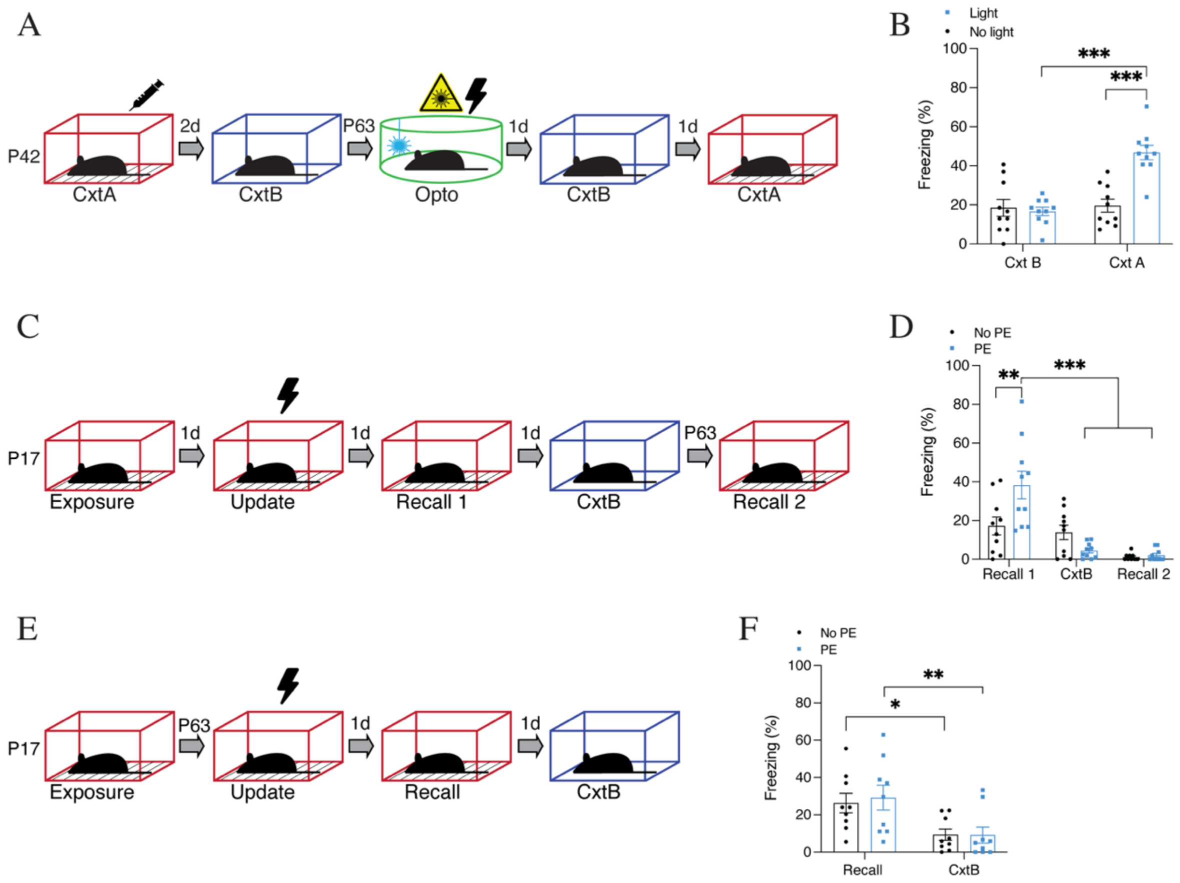

Fig. S11. Infant mice can form context-specific memories that can be behaviorally updated. (**A**) Behavioral schedule for artificial updating of an adult engram in Ai32-FosTRAP mice. (**B**) Freezing levels during recall in context B and context A (n=10). Experimental light group froze significantly more in context A (*P < 0.001*). (**C**) Behavioral schedule for updating paradigm in C57BL/6J infant (P17) mice. (**D**) Freezing levels (n=10) during recall tests at P19 (Recall 1), P20 (CxtB), and P63 (Recall 2). Infant mice that were pre-exposed (PE) to context A froze significantly (*P < 0.01*) more during recall 1. (**E**) Behavioral schedule for updating paradigm when update occurs after the infantile amnesia period. (**F**) Freezing levels (n=9) during recall tests. *P < 0.05*, *P < 0.01*, *P < 0.001* calculated by (**B**, **D**, **F**) two-way ANOVA with Bonferroni post hoc tests. Data presented as ±SEM.

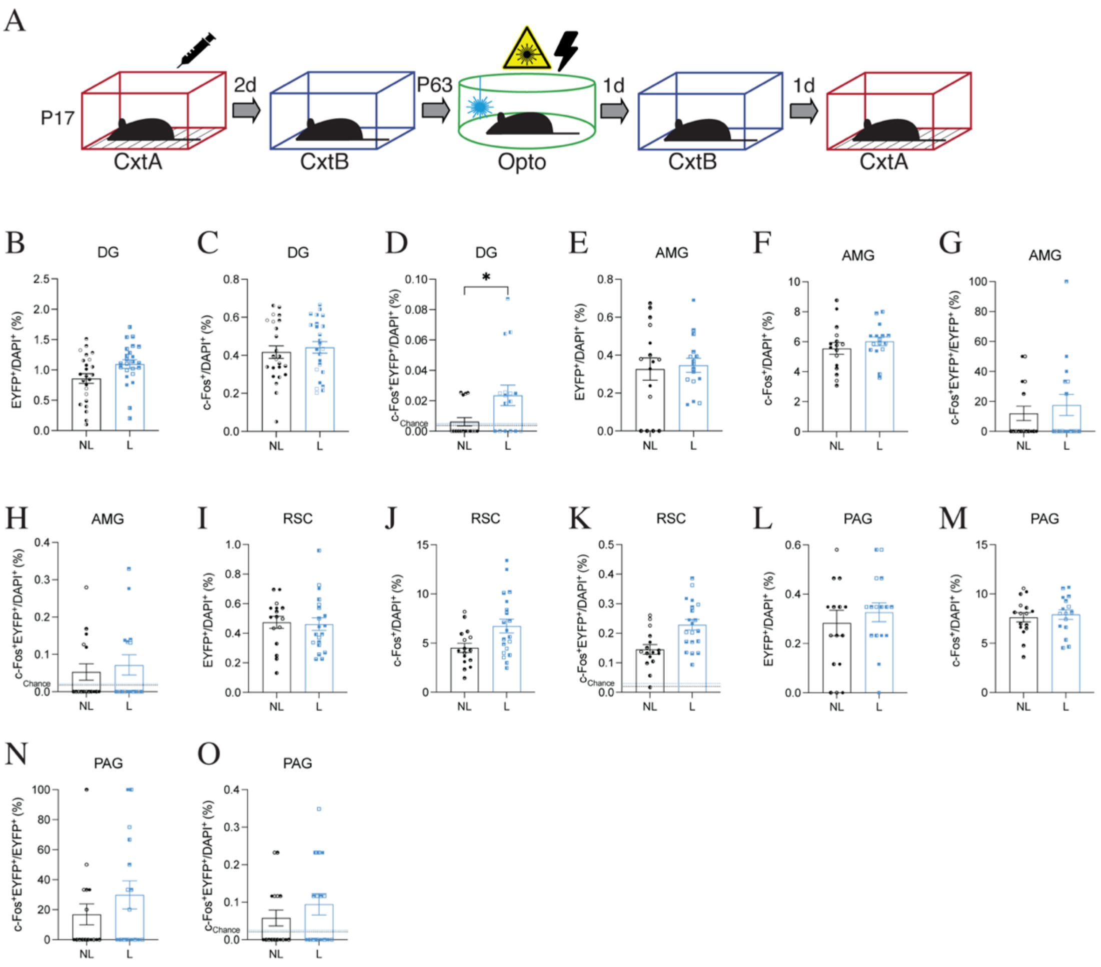

Fig. S12. Artificial updating of infant engram cells in adult mice results in an above-chance level of engram reactivation in the DG, AMG, RSC, and PAG.

(**A**) Behavioral paradigm. (**B-O**) Histological analysis of cell counts after context A recall in adult Ai32-FosTRAP mice. Percentage of (**B**) ChR2-EYFP$^+$ and (**C**) c-Fos$^+$ cells in the DG (N=6, n=4). No light (NL), Light (L). (**D**) Percentage of DAPI cells positive for both ChR2-EYFP and c-Fos. The experimental light group showed a significant increase ($p < 0.05$) in engram reactivation in the DG. Percentage of (**E**) ChR2-EYFP$^+$ and (**F**) c-Fos$^+$ cells in the AMG (N=4, n=4). Engram reactivation in the AMG as a percentage of (**G**) ChR2-EYFP$^+$ cells and (**H**) DAPI$^+$cells. Percentage of (**I**) ChR2-EYFP$^+$ and (**J**) c-Fos$^+$ cells in the RSC (N4-5, n=4). (**K**) Percentage of DAPI cells positive for both ChR2-EYFP and c-Fos. Percentage of (**L**) ChR2-EYFP$^+$ and (**M**) c-Fos$^+$ cells in the PAG (N=4, n=4). Engram reactivation in the PAG as a percentage of (**N**) ChR2-EYFP$^+$ cells and (**O**) DAPI$^+$ cells. $P < 0.05$, $P < 0.01$, $P < 0.001$ calculated by (**B-O**) nested Student t-test. Data presented as ±SEM.

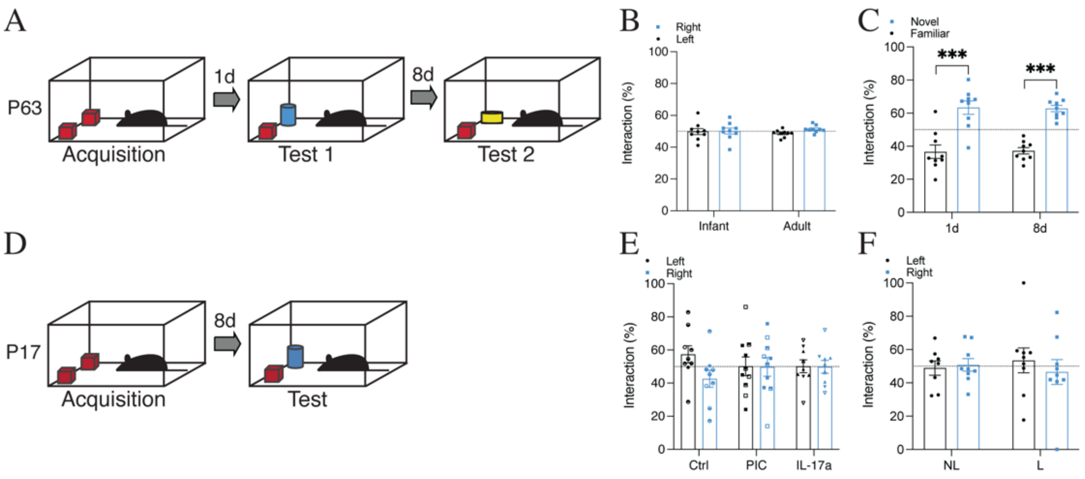

Fig. S13. Adult mice retain an object memory 8 days after training.

(**A**) Behavioral schedule for novel object recognition task in adult C57BL/6J male mice. (**B**) Object interaction during acquisition in infant (P17) (n=10) and adult (P63) (n=9) mice. (**C**) Object interaction (%) during novel object recognition test in adult mice (n=9). Mice spend significantly more time exploring the novel object when tested 1 d ($p < 0.001$) or 8 d ($p < 0.001$) after acquisition. (**D**) Behavioral schedule for novel object recognition task in infant (P17) MIA offspring. (**E**) Object interaction (%) during acquisition in infant (P17) C57BL/6J MIA male offspring (n=9-10). (**F**) Object interaction (%) during acquisition in infant (P17) male Ai32-FosTRAP mice (n=8-9). No light (NL), light (L). $P < 0.05$, $P < 0.01$, $P < 0.001$ calculated by (**E**) nested ANOVA or **(B, C, F**) two-way ANOVA with Bonferroni post hoc tests. Data presented as ±SEM.

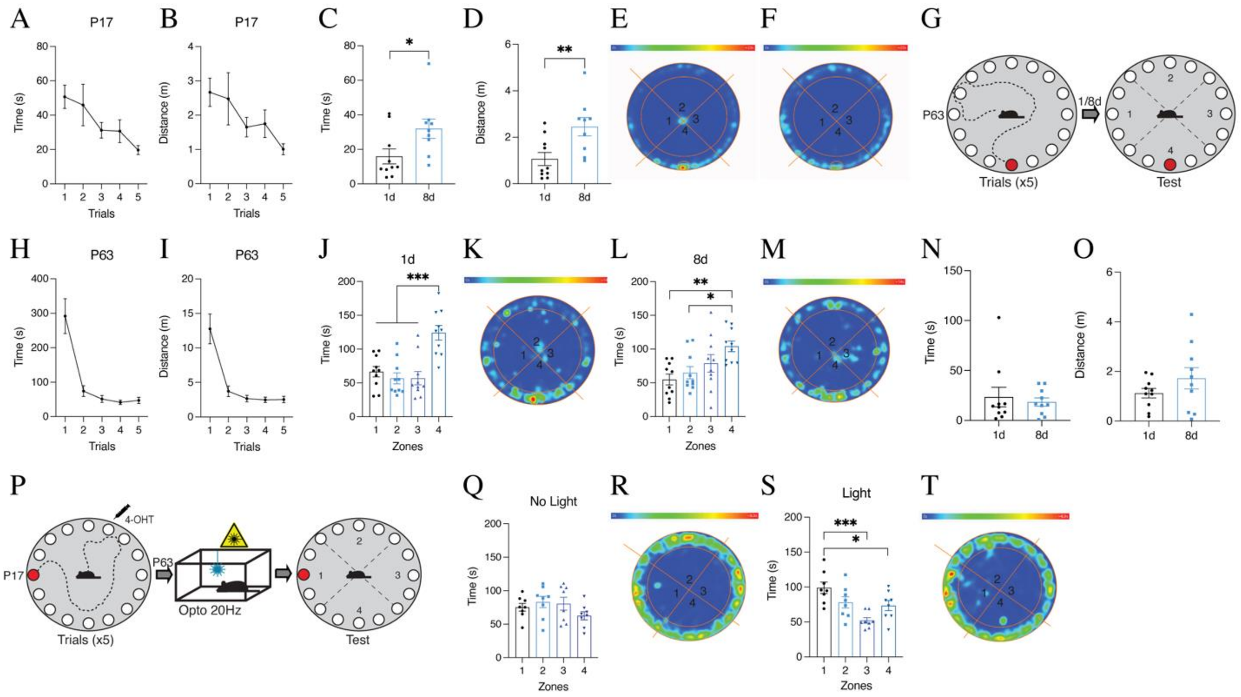

Fig. S14. Adult mice retain both a spatial memory for the location of the escape hole on the Barnes maze task 8 days after training.

(**A**) Time (s) and (**B**) distance (m) taken by infant C57BL/6J mice to reach the escape hole during each trial (n=18-20). (**C**) Time (s) and (**D**) distance (m) taken to first reach where the escape hole should be located during probe tests (n=9-10). (**E**, **F**) Heat map analysis of probe test (**E**) 1 d or (**F**) 8 d after training. (**G**) Behavioral schedule for the Barnes maze in adult (P63) C57BL/6J male mice. (**H**) Time (s) and (**I**) distance (m) taken to reach the escape hole during each trial (n=20). (**J**-**M**) Time spent in each zone during probe test (**J**, **K**) 1 d (*P < 0.001*) or (**L**, **M**) 8 d (*P < 0.05*, *P < 0.01*) after training. (**N**) Time (s) and (**O**) distance (m) taken to first reach where the escape hole should be located during probe tests. (**P**) Behavioral schedule for DG optogenetic reactivation of an infant encoded engram for the Barnes maze. (**Q**, **S**) Time spent in each zone during the test (n=8-9). (**R**, **T**) Heat map analysis of probe test. *P < 0.05, P < 0.01, P < 0.001* calculated by (**C**, **D**, **N**, **O**) Student t-test or (**J**, **L**, **Q**, **S**) ANOVA with Bonferroni post hoc tests. Data presented as ±SEM.

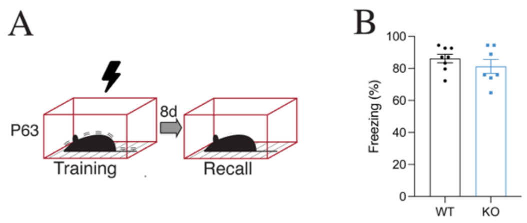

Fig. S15 *Il17a* KO adult mice show natural memory retention for contextual fear memory when tested 8 d after training.

(**A**) Behavioral schedule for CFC in *Il17a* KO adult (P63) mice. The black lightning symbol represents foot shocks. (**B**) Freezing levels (%) during recall in context A. $P < 0.05$, $P < 0.01$, $P < 0.001$ calculated by (**B**) Student t-test. Data presented as ±SEM.

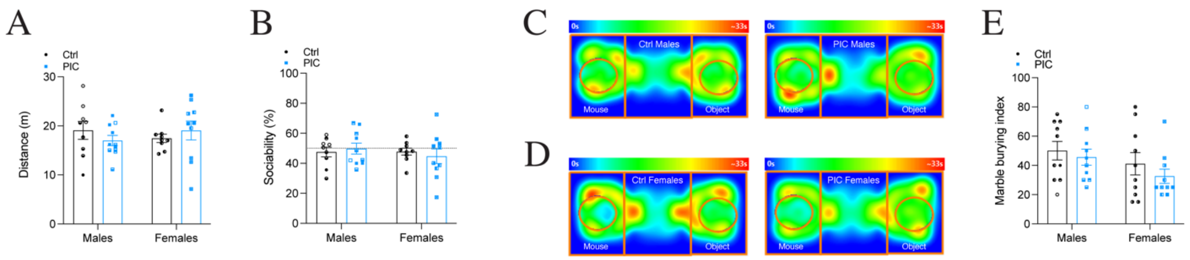

Fig. S16. *Il17a* KO MIA offspring do not show social interaction deficits or repetitive behavior. (**A**) Total distance (m) traveled during social interaction task in adult (P63) male and female *Il17a* KO MIA offspring (n=9-10). (**B**) Social interaction index (%) of adult male and female *Il17a* KO MIA offspring. (**C**, **D**) Heat map analysis of time spent in each chamber for (**C**) male and (**D**) female *Il17a* KO adult MIA offspring. (**E**) Number of marbles buried during marble burying task (n=10). $P < 0.05$, $P < 0.01$, $P < 0.001$ calculated by (**A**, **B, E**) nested ANOVA with Bonferroni post hoc tests. Data presented as ±SEM.

Figure	Sex	Strain	Injection	Compound	Cohort	Litters	n
1E	M	C57BL/6J	E12.5	PBS	Ctrl	2	10
	M	C57BL/6J	E12.5	poly (I:C)	PIC	5	10
	M	C57BL/6J	E12.5	rmIIL-17A	IL-17a	4	10
1F	M	C57BL/6J	E12.5	PBS	Ctrl	2	10
	M	C57BL/6J	E12.5	poly (I:C)	PIC	5	10
	M	C57BL/6J	E12.5	rmIIL-17A	IL-17a	4	10
IG	M	C57BL/6J	E12.5	PBS	Ctrl NS	3	10
	M	C57BL/6J	E12.5	PBS	Ctrl S	3	10
	M	C57BL/6J	E12.5	poly (I:C)	PIC NS	3	9
	M	C57BL/6J	E12.5	poly (I:C)	PIC S	4	10
	M	C57BL/6J	E12.5	rmIIL-17A	IL-17a NS	4	9
	M	C57BL/6J	E12.5	rmIIL-17A	IL-17a S	4	10
2L-N	M	Ai32FosTRAP	E12.5	PBS	Ctrl	4	5
	M	Ai32FosTRAP	E12.5	poly (I:C)	PIC	3	5
2P-R	M	Ai32FosTRAP	E12.5	PBS	Ctrl	4	4
	M	Ai32FosTRAP	E12.5	poly (I:C)	PIC	2	4
4D	M	C57BL/6J	E12.5	PBS	Ctrl	4	9
	M	C57BL/6J	E12.5	poly (I:C)	PIC	3	10
	M	C57BL/6J	E12.5	rmIIL-17A	IL-17a	4	9
4L	M	C57BL/6J	E12.5	PBS	Ctrl	5	10
4M	M	C57BL/6J	E12.5	poly (I:C)	PIC	5	9
4N	M	C57BL/6J	E12.5	rmIIL-17A	IL-17a	3	11
5C	M	Il17a-/-	E12.5	PBS	Ctrl 1d	4	9
	M	Il17a-/-	E12.5	PBS	Ctrl 8d	3	9
	M	Il17a-/-	E12.5	poly (I:C)	PIC 1d	4	8
	M	Il17a-/-	E12.5	poly (I:C)	PIC 8d	4	8
5F	M	C57BL/6J	P3, P7, P14	PBS	Ctrl	5	11
	M	C57BL/6J	P3, P7, P14	poly (I:C)	PIC	6	10
5H	M	C57BL/6J	E12.5, P17	poly (I:C), PBS	PIC+PBS	5	9
	M	C57BL/6J	E12.5, P17	poly (I:C), rmIIL-17A	PIC+IL-17a	5	8
5I	M	C57BL/6J	E12.5, P25	poly (I:C), PBS	PIC+PBS	7	11
	M	C57BL/6J	E12.5, P25	poly (I:C), rmIIL-17A	PIC+IL-17a	7	12
S2A, B	M	C57BL/6J	E12.5	PBS	Ctrl	2	10
	M	C57BL/6J	E12.5	poly (I:C)	PIC	5	10
	M	C57BL/6J	E12.5	rmIIL-17A	IL-17a	4	10
S2C-E	F	C57BL/6J	E12.5	PBS	Ctrl	5	10
	F	C57BL/6J	E12.5	poly (I:C)	PIC	4	10
	F	C57BL/6J	E12.5	rmIIL-17A	IL-17a	4	10
S2F	F	C57BL/6J	E12.5	PBS	Ctrl	4	8
	F	C57BL/6J	E12.5	poly (I:C)	PIC	4	9
	F	C57BL/6J	E12.5	rmIIL-17A	IL-17a	4	10
S2G	M	C57BL/6J	E12.5	PBS	Ctrl	4	10
	M	C57BL/6J	E12.5	poly (I:C)	PIC	5	10
	M	C57BL/6J	E12.5	rmIIL-17A	IL-17a	4	10
S2H	F	C57BL/6J	E12.5	PBS	Ctrl	5	10
	F	C57BL/6J	E12.5	poly (I:C)	PIC	3	9
	F	C57BL/6J	E12.5	rmIIL-17A	IL-17a	4	10
S2I	F	C57BL/6J	E12.5	PBS	Ctrl	5	9
	F	C57BL/6J	E12.5	poly (I:C)	PIC	3	9
	F	C57BL/6J	E12.5	rmIIL-17A	IL-17a	5	9
S2J	M	C57BL/6J	E12.5	poly (I:C)	PIC P25	4	10
	M	C57BL/6J	E12.5	poly (I:C)	PIC P32	4	13
S2K	M	C57BL/6J	E12.5	PBS	Ctrl	3	10
	M	C57BL/6J	E12.5	poly (I:C)	PIC	4	10
	F	C57BL/6J	E12.5	PBS	Ctrl	5	9
	F	C57BL/6J	E12.5	poly (I:C)	PIC	3	9
S7	F	Ai32FosTRAP	E12.5	PBS	Ctrl	3	4
	F	Ai32FosTRAP	E12.5	poly (I:C)	PIC	3	4
S13E	M	C57BL/6J	E12.5	PBS	Ctrl	4	9
	M	C57BL/6J	E12.5	poly (I:C)	PIC	3	10
	M	C57BL/6J	E12.5	rmIIL-17A	IL-17a	4	9
S15A, B	M	Il17a-/-	E12.5	PBS	Ctrl	4	9
	M	Il17a-/-	E12.5	poly (I:C)	PIC	4	10
	F	Il17a-/-	E12.5	PBS	Ctrl	2	9
	F	Il17a-/-	E12.5	poly (I:C)	PIC	4	10
S15E	M	Il17a-/-	E12.5	PBS	Ctrl	5	10
	M	Il17a-/-	E12.5	poly (I:C)	PIC	4	10
	F	Il17a-/-	E12.5	PBS	Ctrl	2	10
	F	Il17a-/-	E12.5	poly (I:C)	PIC	4	10

Table S1. Record of litters used for each immune activation experiment.

Maternal Immune Activation Model Reporting Guidelines Checklist

<table>
<tr><th>ARRIVE Reporting Guideline & Recommendation</th><th>Arrive Item</th><th>MIA Model Specific Reporting Recommendation
Please complete this chart for each point outlined below. If not applicable, write N/A</th></tr>
<tr><td>Study design
➢ Overview of immune activation issues

For each experiment, give brief details of the study design including:
a. The number of experimental and control groups.
b. Any steps taken to minimize the effects of subjective bias when allocating animals to treatment (e.g. randomization procedure) and when assessing results (e.g. if done, describe who was blinded and when).
c. The experimental unit (e.g. a single animal, group or cage of animals).

A time-line diagram or flow chart can be useful to illustrate how complex study designs were carried out.</td><td>6</td><td>MIA Specific Reporting:
a. General need for improved reporting in MIA model methods + reporting pilot data
○ Details on pilot data:

Mice were randomly assigned to experimental groups with littermate controls or otherwise relevant controls. During behavioral analysis, investigators were blinded to the treatment conditions for all experimental groups. Histological analysis was conducted individually for each mouse using nested analysis, where > 4 slices per mouse were analyzed. The same approach was applied to maternal immune activation experiments where nested analysis was performed per litter. Throughout all experiments analysis was conducted blindly.

For number of experimental and control groups please refer to Table 1 in supplementary materials.</td></tr>
<tr><td>Experimental procedures
➢ Compounds
➢ Validation measures

For each experiment and each experimental group, including controls, provide precise details of all procedures carried out. For example:
a. How (e.g. drug formulation and dose, site and route of administration, anaesthesia and analgesia used [including monitoring], surgical procedure, method of euthanasia). Provide details of any specialist equipment used, including supplier(s).
b. When (e.g. time of day).
c. Where (e.g. home cage, laboratory, water maze).
d. Why (e.g. rationale for choice of specific anaesthetic, route of administration, drug dose used).</td><td>7</td><td>Provide details of:
a. Compounds – source, vehicle, preparation/storage, administration route, volume administered, whether anesthetics were used at time of immune challenge.
○ Name of compound: Polyinosinic:polycytidylic acid or rmIL-17A
○ Catalogue number: InvivoGen 31852-29-6 or ImmunoTools 12340174
○ Lot number: 5934-42-01/2, 5934-43-01/2/3/4/5, 5934-44-01
○ Vehicle control used: Phosphate buffer solution
○ Route of administration: subcutaneous
○ Volume administered: 20mg/kg (200ul) or 50ug/kg (200ul)
○ Storage conditions: -20°C
○ Anesthetic (type, dose, duration) used: n/a
b. Housing variables at injection - temperature of room at injection time, cage change at time of injection or not
○ Light cycle of animal housing room: 07:00-19:00 (12 h)
○ Time of day of injection: 2-3pm
○ Room temperature at injection time: 20-21°C
○ Did a cage change occur at time of injection: No</td></tr>
<tr><td></td><td></td><td>c. Validation of immune activation – behavior, physiological indices and/or cytokine data, including pilot dosing data
○ Method used to verify immune activation:

Blood serum IL-17a cytokine levels were measured by ELISA from female mice 24 h after subcutaneous administration of either Poly IC or PBS control.

d. Validation of gestational timing – vaginal plug, estrous cycle, weight gain
○ Method of validating gestational timing:
Weight gain: Dams were time mated for one night and weighed at E0 and then again at E11.

Additional comments:
N/A</td></tr>
<tr><td>Experimental animals
➢ Species/strain/vendor

a. Provide details of the animals used, including species, strain, sex, developmental stage (e.g. mean or median age plus age range) and weight (e.g. mean or median weight plus weight range).
b. Provide further relevant information such as the source of animals, international strain nomenclature, genetic modification status (e.g. knock-out or transgenic), genotype, health/immune status, drug or test naïve, previous procedures, etc.</td><td>8</td><td>Provide details of:
a. Species – considerations for appropriate species (mouse, rat, non human primate, other)
○ Species: mouse

b. Strain – variability in strain can influence model
○ Strain: C57BL/6JOlaHsd, Ai32(RCL-ChR2(H134R)/EYFP or Il17a KO

c. Maternal/Offspring Physiological Variables at time of immune challenge – age, body weight
○ Maternal Age at challenge: 8-12 weeks
○ Maternal Body weight: 24-28g (dependent on pregnant dams litter size)
○ Offspring Age at challenge: E12.5
○ Offspring Sex: Both males and females tested
○ Offspring Body weight: n/a

d. Vendor – even within the same strain, vendor can influence endpoints
○ Vendor: Jackson Labratory
○ Location of Vendor: USA
○ Room/area where animals originated from: Refer to comments</td></tr>
</table>

		Additional Comments:<br>Mouse strains were obtained from JAX and rederived in to the facility. Each line was then bred within the facility and never underwent previous procedures.
**Housing and husbandry**<br>➢ Cage, ventilation, bedding, enrichment<br><br>Provide details of:<br>a. Housing (type of facility e.g. specific pathogen free [SPF]; type of cage or housing; bedding material; number of cage companions; tank shape and material etc. for fish).<br>b. Husbandry conditions (e.g. breeding program, light/dark cycle, temperature, quality of water etc for fish, type of food, access to food and water, environmental enrichment).<br>c. Welfare-related assessments and interventions that were carried out prior to, during, or after the experiment.	9	Provide details of:<br>a. Caging systems<br>○ *At breeding*<br>Material of cage: GM500 cages<br>Cage dimensions: Floor area: 501 cm2<br>○ *After parturition*<br>Material of cage: GM500 cages<br>Cage dimensions: Floor area: 501 cm2<br>○ *At weaning*<br>Material of cage: GM500 cages<br>Cage dimensions: Floor area: 501 cm2<br><br>b. Animal Holding room<br>○ Temperature in room: 20-21°C<br>○ Humidity in room: 50-55%<br>○ Ventilation system: IVC ventilation<br>○ Specific pathogen free [SPF]: YES<br>○ Are males & females housed in the same or separate rooms:<br>Housed in same room after weaning<br>c. Bedding exchanges/bedding type<br>○ Type of cage bedding used: SAFE® Select Bedding Rettenmaier<br>○ Frequency of cage changes per week<br>*during gestation:* 1<br>*during neonatal period:* 0 in 1st week, then every second week<br>*following weaning:* every second week<br><br>d. Breeding - bred on site or timed pregnant, how many different sires (are the same fathers breeding with both experimental and control dams)<br>Breeding location: on site
		○ Gestational age at shipping: N/A<br>○ Biological age of dams (if not listed in Section 8c): 8-12 weeks<br>○ Number of Dams bred: 160<br>○ How many times have dams been mated previously: 0 (virgins used)<br>○ How many times did the dams mate and not become pregnant: 1-2<br>○ Are the dams primiparous or multiparous? All dams are multiparous<br>○ What was the frequency of maternal handling during the gestational/neonatal period (e.g. cage cleanings, weighing, blood collection manipulations): weighing at E11, change cage at E13<br>○ Biological age of sires: 7-12<br>○ Number of sires bred: 55<br>○ How many times have sires been mated previously: 2-3<br>○ How many times did the sires mate successfully (e.g. mating resulted in pregnancy, full term birth): 2-3<br>○ If bred previously, what was the interval between mating times:<br>○ Are sires matched to experimental and control dams: No<br>○ Describe the mating design (1:1, 1:2 etc): paired 1:1 for one night<br><br>e. Social enrichment – number of cage companions<br>○ Number of cage companions prior to breeding: 1-5<br>○ Gestational age when dam separated for parturition: E13<br>○ Number of cage companions at weaning: 1-5<br><br>f. Physical enrichment – describe enrichment devices, and when enrichment is in the cage (removed when pups born? Or present throughout study), does the enrichment type change? How frequently?<br>○ Describe what type of enrichment devices (and how many) are included in cage/housing room:<br>Enrichment stays consistent throughout experiment. Each cage contains one tunnel tube and bedding.

		○ Does enrichment type/access change across study? No<br>○ If so, when does enrichment type/access change (e.g. enrichment removed prior to parturition and replaced in late neonatal period):<br>N/A<br><br>Additional Comments:<br>Only virgin dams were used for mating purposes and were not mated until 8 weeks of age to ensure survival of the litter. Dams were time mated 1:1 for one night and then placed back in their homecage. Dams were housed with cage mates until E13 where they were transfered to a new cage with one other dam (cage mate). It was important to ensure that the cage mate had not been time mated to prevent discrepancies with P0 of the litter.
**Sample size**<br>➢ Litter versus offspring<br><br>a. Specify the total number of animals used in each experiment, and the number of animals in each experimental group.<br>b. Explain how the number of animals was arrived at. Provide details of any sample size calculation used.<br>c. Indicate the number of independent replications of each experiment, if relevant.	10	Provide details of:<br>a. Maternal N vs offspring N<br>○ What is the total number of dams/litters included in the study: 160<br>○ What is the total number of offspring per litter included the study: 2-6<br>b. Litter size and sex distribution<br>○ What size was each litter maintained at: 6<br>○ What age did culling take place at: P10-P12<br>○ How many males and females were maintained in each litter: N/A<br>c. Cross fostering<br>○ Did cross fostering occur: No<br>○ If so, at what age did cross fostering occur: N/A<br><br>Additional Comments:
**Allocating animals to experimental groups**<br><br>a. Give full details of how animals were allocated to experimental groups, including randomization or matching if done.<br>b. Describe the order in which the animals in the different experimental groups were treated and assessed.	11	a. How many offspring per litter were used in each measure: refer to S.Table 1<br>b. Randomization/Matching procedures<br>○ What procedures were used to assign animals to groups:<br>Litters from damns that were injected on the same day were counter balanced across groups.<br><br>c. Sex as a biological variable (behavioral and physiological outcomes)<br>○ Were both males and females evaluated in each behavioral and physiological outcome: Yes<br><br>Additional Comments:
**Experimental outcomes**<br>➢ Behavioral testing<br>➢ Physiological endpoints<br><br>Clearly define the primary and secondary experimental outcomes assessed (e.g. cell death, molecular markers, behavioral changes).	12	a. Maternal behavior and pup interactions<br>○ If maternal care was evaluated, were there differences following immunogen challenge (if so, please briefly describe):<br>N/A<br>b. Age(s) of offspring at behavioral testing/physiological evaluation endpoints:<br>CFC: P17 and recall at P18, P25 or P32 ; Social interaction and marble burying: P63-P65<br>Engram labeling: P17 ; Perfusions P25<br>c. Order of testing (e.g. behavioral test order)<br>○ Were animals evaluated in a counter-balanced order in terms of:<br>*presentation of tests to each animal:* Yes<br>*order of experimental/control groups run through each test:* Yes<br>○ What was the inter-test interval if a single animal underwent a battery of tests:<br>38 days between CFC and sociability test; 1 day in between sociability and marble burying tests.

		Additional Comments:
**Statistical methods**<br><br>a. Provide details of the statistical methods used for each analysis.<br>b. Specify the unit of analysis for each dataset (e.g. single animal, group of animals, single neuron).<br>c. Describe any methods used to assess whether the data met the assumptions of the statistical approach.	13	a. Unit of analysis for each data set<br>○ Is the unit (n) of each analysis based on number of litters, or number of animals used per group:<br><br>For behavioral analysis, results were analyzed per litter using nested t-test or nested ANOVA followed by Bonferroni post hoc test. For cell counting analysis results were analyzed per mouse using nested t-test or nested ANOVA followed by Bonferroni post hoc test. Results were considered significant if $P<0.05$.
**Other Disclosures**		Please make note of any other extraneous variables that you would like to report (e.g. fire alarms, construction, temporary relocations, other variables that you think we should be considering in our studies etc.):<br><br>N/A

Table S2. Maternal immune activation model reporting guidelines checklist (66).
